# Thermodynamic Study of Oxidovanadium(IV) with Kojic Acid Derivatives: A Multi-Technique Approach

**DOI:** 10.3390/ph14101037

**Published:** 2021-10-12

**Authors:** Rosita Cappai, Guido Crisponi, Daniele Sanna, Valeria Ugone, Andrea Melchior, Eugenio Garribba, Massimiliano Peana, Maria Antonietta Zoroddu, Valeria Marina Nurchi

**Affiliations:** 1 Dipartimento di Scienze della Vita e dell’Ambiente, Università di Cagliari, 09042 Cagliari, Italy; crisponi@unica.it; 2 Istituto di Chimica Biomolecolare, Consiglio Nazionale delle Ricerche, Trav. La Crucca 3, 07100 Sassari, Italy; daniele.sanna@cnr.it (D.S.); valeria.ugone@cnr.it (V.U.); 3 DPIA, Laboratorio di Scienze e Tecnologie Chimiche, Università di Udine, Via del Cotonificio 108, 33100 Udine, Italy; andrea.melchior@uniud.it; 4 Dipartimento di Chimica e Farmacia, Università di Sassari, via Vienna 2, 07100 Sassari, Italy; garribba@uniss.it (E.G.); peana@uniss.it (M.P.); zoroddu@uniss.it (M.A.Z.)

**Keywords:** oxidovanadium(IV), kojic acid, potentiometry, UV-visible spectrophotometry, EPR spectroscopy, ESI-MS spectrometry, DFT calculations

## Abstract

The good chelating properties of hydroxypyrone (HPO) derivatives towards oxidovanadium(IV) cation, V^IV^O^2+^, constitute the precondition for the development of new insulin-mimetic and anticancer compounds. In the present work, we examined the V^IV^O^2+^ complex formation equilibria of two kojic acid (KA) derivatives, L4 and L9, structurally constituted by two kojic acid units linked in position 6 through methylene diamine and diethyl-ethylenediamine, respectively. These chemical systems have been characterized in solution by the combined use of various complementary techniques, as UV-vis spectrophotometry, potentiometry, NMR and EPR spectroscopy, ESI-MS spectrometry, and DFT calculations. The thermodynamic approach allowed proposing a chemical coordination model and the calculation of the complex formation constants. Both ligands L4 and L9 form 1:1 binuclear complexes at acidic and physiological pHs, with various protonation degrees in which two KA units coordinate each V^IV^O^2+^ ion. The joined use of different techniques allowed reaching a coherent vision of the complexation models of the two ligands toward oxidovanadium(IV) ion in aqueous solution. The high stability of the formed species and the binuclear structure may favor their biological action, and represent a good starting point toward the design of new pharmacologically active vanadium species.

## 1. Introduction

Vanadium compounds show a wide variety of pharmacological properties in humans, among which antiparasitic, antiviral, antibacterial and, particularly, antidiabetic and anticancer action [1,2,3,4,5,6,7]. A number of studies have shown that vanadium compounds favor glucose intake into cells, lowering the level of glucose in blood; vanadium compounds, with respect to insulin, present the advantage of being orally active [8,9,10,11]. A quantity of V^IV^ complexes have been synthesized and characterized [12], while one of them, formed by a hydroxypyrone (HPO) derivative, V^IV^O(ethylmaltolato)_2_, has passed tests in phase 1 and phase 2 clinical trials in Canada and in the USA [13]. These vanadium compounds should be neutral and possess the proper lipophilicity to cross easily the cell membranes. Nevertheless, Kiss, Sakurai and coworkers, based on speciation studies, remarked that the neutral bis-chelated complexes are not stable, and at acidic pH values they can decompose with the formation of ionic complexes or free metal ion of low absorption ability [12,14,15].

In recent years, vanadium compounds have emerged as useful anticancer agents because of desirable properties for chemotherapeutic reagents, displaying higher selectivity, low toxicity, greater reactivity, as well as anti-metastatic activity [16,17,18]. Among the new potential anticancer agents, the bis-chelated complex formed with the well-known iron chelator deferiprone, the 1,2-dimethyl-3-hydroxy-4(1*H*)-pyridinone, is active against malignant melanoma cells and causes apoptosis and cell cycle block [19,20]. It is noteworthy that polynuclear metal complexes often have a cytotoxicity higher than mononuclear species, as was reported for Au, Pd, Pt, and Cu complexes [21,22,23,24,25,26,27,28].

One of main requirements for new potential V^IV^O^2+^ drugs is the thermodynamic stability to survive enough in the serum, enter intact into the cells and release the active species only in the cytosol. In fact, it has been recently demonstrated that the cellular uptake of vanadium compounds is reduced upon transferrin binding, and that this interaction may inhibit, instead of promoting, their biological and pharmacological activity [29].

Based on the above considerations, in this study we have synthesized ligands for V^IV^O^2+^ complexation that combine different advantages, among which the formation of polynuclear complexes and their high stability are the most noteworthy. The use as a ligand of kojic acid (KA), a natural, non-toxic and low-cost product of large use in food and cosmetic industries, their good chelating properties towards V^IV^O^2+^ and the simple synthesis constitute further advantages.

**Figure 1:**
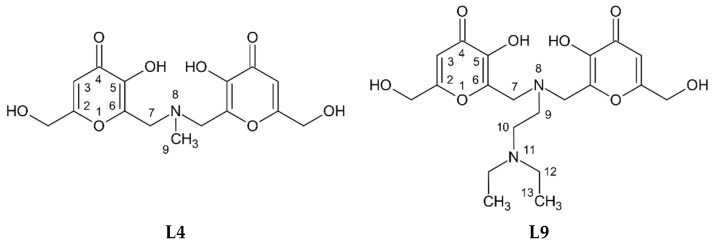
Molecular structure of L4 and L9 ligands.

Previously, we investigated the interaction between V^IV^O^2+^ and three linear KA derivatives, in which two KA units are linked in position 2 by diamines of different length; these ligands, depending on the length of the linker, form V^IV^O^2+^ complexes with various structure and protonation degree [30]. In the current work, we present a potentiometric-spectrophotometric study supported by EPR, NMR, ESI-MS measurements, and DFT calculations of V^IV^O^2+^ complexation with two KA derivatives, named L4 and L9 (Figure 1). In these ligands, the two KA units are linked in position 6 by methylamine and diethyl-ethylenediamine respectively, showing an orientation of KA units opposite to that in the ligands previously studied [30]. The complex formation equilibria with other metal ions (Fe^3+^, Al^3+^, Cu^2+^ and Zn^2+^) has been already studied; in the case of the hard Fe^3+^ metal ion, the formation of binuclear Fe_2_L_2_ complexes was observed, with an increase of stability of more than four orders of magnitude with respect to the parent KA ligand [31,32,33,34].

After the characterization of the systems in aqueous solution, biological tests to evaluate the antidiabetic and cytotoxic potential of these vanadium complexes will be carried out in a next research step.

## 2. Experimental

### 2.1. Reagents

NaOH, NaCl, HCl, kojic acid, and VOSO_4_·3H_2_O were Sigma-Aldrich (Milano, Italy) products, and were used without any further purification. L4 and L9 ligands were synthetized as reported in Refs. [31,33]. Carbonate free 0.1 M NaOH solution was prepared as previously described [35]. Oxidovanadium(IV) sulphate solution ~0.1 M was prepared weekly, acidified with a stoichiometric amount of HCl to prevent hydrolysis and standardized by redox titration as reported by Berto et al. [36]. All solutions were prepared using grade A glassware and ultrapure water (conductivity < 0.1 μS).

### 2.2. Solution Equilibrium Studies

The complex formation equilibria were studied at 25 °C and 0.1 M NaCl ionic strength by combined potentiometric-spectrophotometric titrations at 1:1, 1:2 and 1:4 V^IV^O^2+^:ligand molar ratios with a constant ligand concentration of 3.0 × 10^−4^ M. Potentiometric measurements were performed with a dEcotrode plus Metrohm combined glass electrode connected to an 888 Titrando titrator (Metrohm AG, Herisau, Switzerland). The electrode was calibrated daily for hydrogen ion concentration by HCl standard titration with NaOH in the used experimental conditions, and data were analyzed by Gran’s method [37]. Spectrophotometric measurements were performed in the 200–400 nm range with a 0.2 cm fiber optic dip probe connected to an Agilent Cary 60 UV-vis spectrophotometer. Potentiometric and spectrophotometric data were processed by HyperQuad and HypSpec programs, respectively [38,39]. Log β*_pqr_* values refer to the overall equilibria *p*V + *q*H + *r*L ⇆ V*_p_*H*_q_*L*_r_* (electrical charges omitted). During the calculations, the following hydroxido complexes of V^IV^O^2+^ were assumed: [V^IV^O(OH)]^+^ (log β_1–1_ = −5.94), [(V^IV^O)_2_(OH)_2_]^2+^ (log β_2–2_ = −6.95) [40], [V^IV^O(OH)_3_]^−^ (lo gβ_1–3_ = −18.0) and [(V^IV^O)_2_(OH)_5_]^−^ (log β_2–5_ = −22.0) [41,42].

### 2.3. ESI-MS Measurements

The solutions for ESI-MS measurements were prepared by dissolving a weighted amount of the ligand L4 or L9 in a V^IV^O^2+^ solution (1 mM, in LC-MS grade water or MeOH) to have a 1:1 V^IV^O^2+^:ligand molar ratio. Argon was bubbled to avoid the oxidation of V^IV^ to V^V^, and the pH was raised up to 7.0 with ammonium carbonate. Subsequently, the solutions were diluted to 50 µM or 5 µM immediately before recording the mass spectra. Positive-ion mode ESI-MS spectra were recorded with a high-resolution Q Exactive™ Plus Hybrid Quadrupole-Orbitrap™ mass spectrometer (Thermo Fisher Scientific, Milano, Italy). The solutions were infused at a flow rate of 5.00 μL/min into the ESI chamber. Spectra were recorded in the range of *m/z* 80–1200 with a resolution of 140,000. The instrumental conditions were as follows: spray voltage 2300 V, capillary temperature 250 °C, sheath gas 5 (arbitrary units), auxiliary gas 3 (arbitrary units), sweep gas 0 (arbitrary units), probe heater temperature 50 °C. MS/MS spectra were recorded using Normal Collision Energy (NCE) setting in the range of 10–40 and with an *m/z* range of 1.0 around the peak under investigation; ion fragments were detected with a resolution of 17,500. All the mass spectra were analyzed by using Thermo Xcalibur 3.0.63 software (Thermo Fisher Scientific, Milano, Italy).

### 2.4. EPR Experiments

The solutions were prepared by dissolving in ultrapure water obtained from a Millipore Milli-Q Academic purification system (Merck KGaA, Darmstadt, Germany) a weighted amount of VOSO_4_·3H_2_O and L4 or L9 to obtain a metal ion concentration of 1 or 2 mM and a ligand to metal molar ratio of 1 or 2. The solutions were bubbled with argon to avoid oxidation of the metal ion. The pH values of the solution were varied with diluted solution of H_2_SO_4_ and NaOH. To uniformly freeze the solutions and prevent a concentration gradient during freezing, DMSO was added to each sample (5–10%); under these experimental conditions, the binding of DMSO to V^IV^ can be neglected.

EPR spectra were recorded immediately after the preparation of the solutions at 120 K with an X-band Bruker EMX spectrometer equipped with a HP 53150A microwave frequency counter and a variable temperature unit. The microwave frequency was 9.40–9.41 GHz, microwave power was 20 mW (a value close to the saturating condition to maximize signal intensity), time constant was 81.92 ms, modulation frequency 100 kHz, modulation amplitude 0.4 mT, resolution 4096 points.

The EPR spectrum of [(V^IV^O)_2_(L4)_2_(H_2_O)_2_] was simulated with EasySpin software, vers. 5.2.33 [43,44,45].

### 2.5. NMR Experiments

NMR experiments were performed on a Bruker Ascend™ 400 MHz spectrometer equipped with a 5 mm automated tuning and matching broadband probe (BBFO) with z-gradients. The samples for NMR experiments were in the range of 2–4 mM in H_2_O/D_2_O 90/10 *v/v* or in MeOD-d4 solutions and carried out at 298 K in 5 mm NMR tubes at pH 7.4 at different V^IV^O^2+^ ligands molar ratios. The concentration of V^IV^O^2+^ ion was achieved by using a batch of 100 mM deuterated aqueous or MeOD-d4 solutions of oxidovanadium(IV) sulphate freshly prepared prior each set of NMR experiment acquisition. 2D ^1^H-^13^C heteronuclear single quantum coherence (HSQC) spectra were acquired by using a phase-sensitive sequence employing Echo–Antiecho-TPPI gradient selection with a heteronuclear coupling constant JXH = 145 Hz, and shaped pulses for all 180° pulses on f2 channel with decoupling during acquisition; sensitivity improvement and gradients in back-inept were also used. Relaxation delays of 2 s and 90° pulses of about 10 μs were applied in all the experiments.

2D ^1^H-^1^H correlation spectroscopy (COSY) spectra were acquired using gradient pulses for selection with multiple quantum filter according to gradient ratio using pulse program ‘mqsgp1d2’ for setup gradient ratio optimized for artifact suppression. Solvent suppression was achieved by using excitation sculpting with gradients. All NMR data were processed with TopSpin (Bruker Instruments, Billerica, MA, USA) software and analyzed by Sparky 3.11 and MestRe Nova 6.0.2 (Mestrelab Research S.L., Santiago de Compostela, Spain) programs.

### 2.6. DFT Calculations

The geometry of [(V^IV^O)_2_(L4)_2_(H_2_O)_2_] complex was optimized and the harmonic frequencies computed with Gaussian 09 software (revision *D.01*) [46] at DFT theory level in aqueous solution describing water with the SMD continuum model of Marenich et al. [47]. The functional B3P86 [48,49] and the basis set 6−311++g(d,p), including diffuse and polarization functions for all the atoms, were used according to the reported procedure [50]. The exchange coupling constant *J* was calculated at the level of theory B3LYP/6-311g with ORCA package [51], using the Heisenberg Hamiltonian $\hat{H}=-J{\hat{S}}_{1}\cdot {\hat{S}}_{2}$, where *S*_1_ and *S*_2_ are the spins on two vanadium(IV) atoms [52]. When *S*_1_ = *S*_2_, *J* can be obtained by the expression *J* = *E*_LS_ − *E*_HS_, with *E*_LS_ and *E*_HS_ energies of the singlet and triplet state; the energy of the low spin state, *E*_LS_, can be determined the broken-symmetry solution, *E*_BS_ [53]. The tensor **A**(^51^V) was calculated using the method developed and implemented into the Gaussian package at the level of theory BHandHLYP/6−311+g(d) following the protocol in the literature [54]. Concerning the algebraic sign of the V hyperfine coupling constants [51], they are negative for V^IV^, but their absolute value is reported in this study. The percent deviation (PD) of the absolute calculated value, |*A*_z_|^calcd^, from the absolute experimental value, |*A*_z_|^exptl^, was obtained as follows: 100 × [(|*A*_z_|^calcd^ − |*A*_z_|^exptl^)/|*A*_z_|^exptl^].

## 3. Results and Discussion

### 3.1. Characterization of the Ligands

The synthesis and the characterization of the ligands L4 and L9 has been previously reported, as well as their solid-state structure [31,32,33,34].

In the present study, mass spectrometry measurements were used to confirm the structure of L4 and L9. The peaks at *m/z* 340.10 and 362.08 can be attributed to the adducts with proton and sodium, [L4+H]^+^ and [L4+Na]^+^ and that at 425.19 to [L9+H]^+^ (Table S1, the Tables and Figures denoted as Snumber are reported in the Supplementary Materials). Further evidence is provided by ESI-MS/MS measurement. With L4, MS/MS spectrum recorded in the range *m/z* = 340.10 ± 0.5 shows two peaks at 155.03 and 186.08, due to the ligand fragmentation (Figure S1); similarly, in the spectrum at *m/z* = 425.19 ± 0.5 of L9, the signals of the fragments C_7_H_7_O_4_, C_8_H_12_O_4_N, C_15_H_18_O_8_N at *m/z* 155.03, 186.07 and 340.10, respectively, coming from the breaking of the various C–N bonds, are detected (Figure S2).

### 3.2. Protonation Equilibria

Table 1 reports the protonation constants of L4 and L9 ligands previously published [31,33].

The ^1^H NMR chemical shift variations during a pH titration of L9 ligand allowed the unambiguous assignment of the first deprotonation step to N8 nitrogen atom on the linker, and the fourth deprotonation to N11 nitrogen atom in the lateral chain [34].

The assignments of the protonation constants to the proper basic groups allow us to comment and to explain the different acid behavior of the two ligands. Starting from the most protonated species, the pK value 4.38 of L4, related to the loss of the proton from the N8 nitrogen atom, is ~4 pK units higher than the corresponding value 0.51 for L9, due to the different charge of the starting molecule (2+ in L9 vs. 1+ in L4). An easier formation of stabilizing hydrogen bonding between the neutral N8 atom and the OH group of one of the KA units further lowers the pK of L9. Similarly, the pK values related to the deprotonation of OH groups in the KA moieties are 0.5–1.0 pK units lower in L9, again depending on the different charge of the starting molecules. The L9 ligand has a further protonated group respect to L4, the N11 atom in the lateral chain, characterized by a pK value 10.81. This high value depends both on the negative charge on the starting molecule, and on the hydrogen bonding between N11 and a phenolate group in one KA unit.

Spectrophotometric titration of L4 and L9 ligands (Figures S3–S6) was carried out to evaluate the absorptivity spectra in the experimental conditions. The spectral behavior observed is the same as KA, but with values of ε almost double.

### 3.3. Oxidovanadium(IV) Complex Formation Equilibria

**Figure 2:**
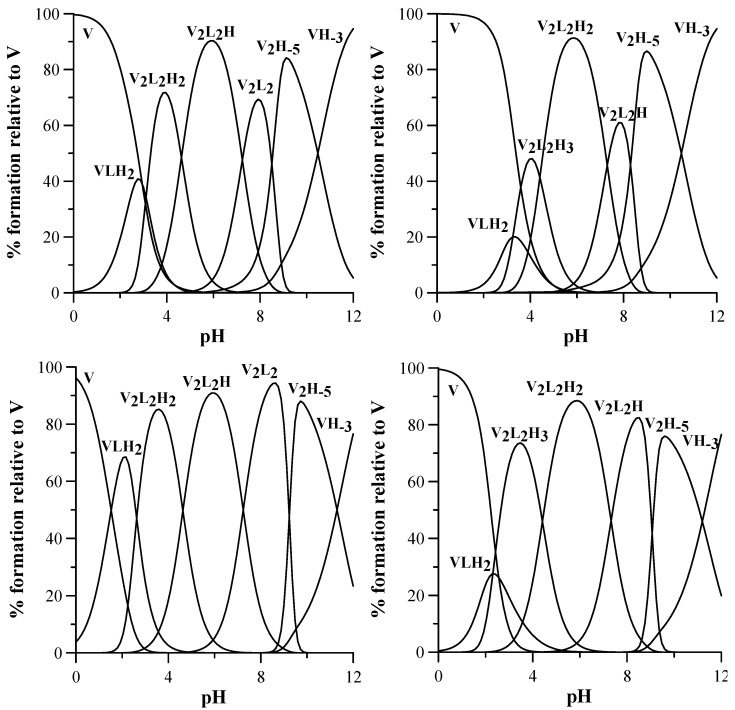
Speciation plots of V^IV^O^2+^-ligand systems (L4 on the left and L9 on the right), calculated with Hyss program [55]. Top: conditions of spectrophotometric titrations, 1:1 V^IV^O^2+^:ligand molar ratio at ligand concentration 3 × 10^−4^ M. Bottom: conditions of EPR measurements 1:2 V^IV^O^2+^:ligand molar ratio at ligand concentration 4 mM. V stands for V^IV^O^2+^, and L for L4 or L9; charges are omitted for simplicity.

The complex formation equilibria involving V^IV^O^2+^ and L4 and L9 were studied by combined potentiometric-spectrophotometric titrations at 1:1, 1:2 and 1:4 V^IV^O^2+^:ligand molar ratios (Figures S7–S14), supported by EPR and ESI-MS measurements. The formed complexes and the related stability constants are reported in Table 2 and the speciation plots in Figure 2.

In the calculation of complex stability constants, the formation of V^IV^O^2+^ hydroxido species was taken into account, assuming the species [V^IV^O(OH)]^+^ with log β_1–1_ = −5.94, [(V^IV^O)_2_(OH)_2_]^2+^ with log β_2–2_ = −6.95, [40] [V^IV^O(OH)_3_]^−^ with log β_1–3_ = −18.0, and finally [(V^IV^O)_2_(OH)_5_]^−^ with log β_2–5_ = −22.0 taken from Komura and Hayashi [41].

**Figure 3:**
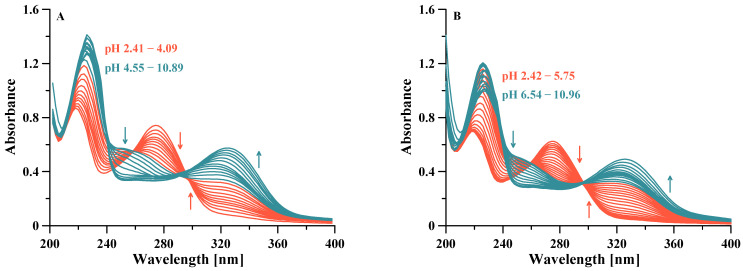
Representative spectra of V^IV^O^2+^-L4 (**A**) and V^IV^O^2+^-L9 (**B**) both at 1:1 molar ratio collected between 200 and 400 nm, l = 0.2 cm at 25 °C, 0.1 M NaCl ionic strength and ligand concentration 3 × 10^−4^ M.

Some representative UV spectra collected at increasing pH values are shown in Figure 3. Potentiometric-spectrophotometric titrations data of V^IV^O^2+^–L4 system at different V^IV^O^2+^: ligand molar ratios (1:1, 1:2 and 1:4) (Figures S7–S10) were fitted assuming the formation of a mononuclear complex [V^IV^OLH_2_]^2+^ at low pH values, in which the V^IV^O^2+^ ion is most likely bound by one KA unit, being the second one and the N8 nitrogen atom still being protonated. The formation of a binuclear complex [(V^IV^O)_2_L_2_H_2_]^2+^starts at pH > 2.5. The first V^IV^O^2+^ ion is bound by two KA units of two different ligands, and the second one by one remaining KA unit, being the fourth KA unit and the N8 nitrogen atom protonated. At increasing pH levels, this complex loses a proton with pK 4.63, presumably from the last KA unit to form [(V^IV^O)_2_L_2_H]^+^ in which both one V^IV^O^2+^ ions are fully coordinated by two KA units. A further proton is lost with pK 7.24, presumably from a coordination water of one V^IV^O^2+^ group. It must be noted that the deprotonation pK of the equatorial water coordinated to vanadium in *cis*-[V^IV^O(KA)_2_(H_2_O)] to give the hydroxido complex *cis*-[V^IV^O(KA)_2_(OH)]^−^ is 8.46 [56]. At pH > 9 the formation of the hydroxido species [V^IV^O(OH)_3_]^−^ and [(V^IV^O)_2_(OH)_5_]^−^ causes the release of the ligands from metal first coordination sphere, and in the corresponding spectra the formation of the deprotonated forms [LH]^−^ and L^2−^ is evident (Figure S8).

A similar complexation scheme is presented by L9, studied by potentiometric-spectrophotometric titrations in the same conditions as above (Figures S11–S14). At low pH values, a mononuclear complex [V^IV^OLH_2_]^2+^ is formed, in which the V^IV^O^2+^ ion is most likely bound by one KA unit being the second and the N11 nitrogen atom still protonated, and N8 deprotonated at this pH values as in the free ligand. At pH > 3, the formation of a binuclear complex [(V^IV^O)_2_L_2_H_3_]^3+^ occurs, in which the first V^IV^O^2+^ group is probably bound by two KA units of two different ligands, and the second V^IV^O^2+^ by one of the remaining KA units, being the second KA protonated, as well as both N11 atoms on the lateral chain of the linker. This complex loses a first proton with pK 4.41, surely not from N11, characterized by a pK 10.81 in the free ligand, and not from a coordinated water, being the pK value too low for such a deprotonation. Therefore, it is likely that the deprotonation occurs on the OH group of KA, forming a complex [(V^IV^O)_2_L_2_H_2_]^2+^ in which both V^IV^O^2+^ ions are fully coordinated by KA units. This complex then loses a further proton with pK 7.30, presumably for the deprotonation of a coordinated water molecule, as happened with L4 (pK 7.24). At pH > 9, the formation of V^IV^O^2+^ hydroxido complexes takes place, as previously observed with L4.

### 3.4. ESI-MS

**Figure 4:**
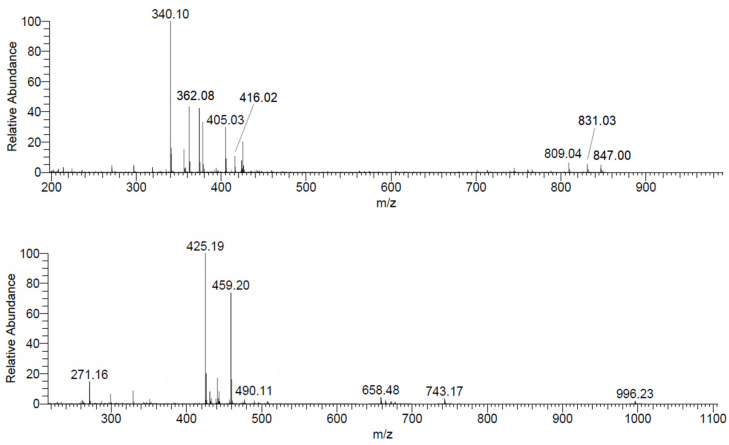
ESI-MS(+) spectrum recorded on the system V^IV^O^2+^-L4 at 1:1 molar ratio (top) and ESI-MS(+) spectrum recorded on the system V^IV^O^2+^-L9 (bottom) at 1:1 molar ratio (LC-MS H_2_O, ligand concentration 50 µM).

The mass spectra recorded on the system V^IV^O^2+^-L4 at 1:1 molar ratio in ultrapure water (Figure 4) confirm the formation of binuclear species in aqueous solution. Different adducts with H^+^, Na^+^ and K^+^ ions were detected, whose *m/z* values are listed in Table 3. The formation of these adducts was confirmed by the comparison between experimental and calculated isotopic pattern of the detected peaks. As an example, comparing the experimental and calculated isotopic pattern (Figures S15 and S16) of the peaks at *m/z* 405.03 and 809.04, the signals can be attributed to [(V^IV^O)_2_(L4)_2_+2H]^2+^ and [(V^IV^O)_2_(L4)_2_+H]^+^, determined also by potentiometric measurements. According to EPR and computational data (Section 3.5 and Section 3.6), this species can be described with the formula [(V^IV^O)_2_(L4)_2_(H_2_O)_2_] with the two V^IV^O^2+^ ions in an octahedral geometry and water ligand in *cis* to the V=O bond, a typical arrangement for KA derivatives [56,57,58]. The lacking detection of two water molecules in the mass spectra is in line with the results in the literature since it has been demonstrated that a weak monodentate ligand can be removed from the metal coordination sphere during the ionization process [59,60,61].

ESI-MS spectra of the system V^IV^O^2+^-L9 were recorded in both aqueous and methanol solution (Figure 4 and Figure S17). In the spectrum recorded in MeOH, besides the signal of the free ligand at *m/z* 425.19, the peaks at *m/z* 490.11 and 979.22 were assigned to the dimeric species [(V^IV^O)_2_(L9)_2_+2H]^2+^ and [(V^IV^O)_2_(L9)_2_+H]^+^ (Table 3) and their composition was confirmed by the isotopic pattern simulations (Figures S18 and S19). These species were also determined by pH-potentiometry. The intensity of the peaks is higher in MeOH than in H_2_O, but this could be related to a better ionization.

In the spectra recorded in ultrapure water, the signals of tri- and tetranuclear species were identified; their structure could be based on V^IV^O^2+^/V^V^O_2_^+^ and V^IV^O^2+^/V^V^_2_O_3_^4+^ groups bridged by three or four (L9)^2−^ anions with an (equatorial–axial) coordination mode. Even though the intensity of the signals is low, various adducts with H^+^ and Na^+^ and different charge, listed in Table 3, were identified. Their elemental composition was confirmed by isotopic pattern calculations (Figure S20). Notably, the fragmentation of these species in the MS/MS spectra results in the formation of [(V^IV^O)_2_(L9)_2_+2H]^2+^ and [(V^IV^O)_2_(L9)_2_+H]^+^. Therefore, it cannot be excluded that in aqueous solution, vanadium complexes with nuclearity higher than two exist in small amount. It cannot be ascertained if the detection of V^V^ moieties is due to the to the partial oxidation in solution of the corresponding V^IV^ complexes or an in-source oxidation process, as already reported in literature [60,62].

### 3.5. EPR

**Figure 5:**
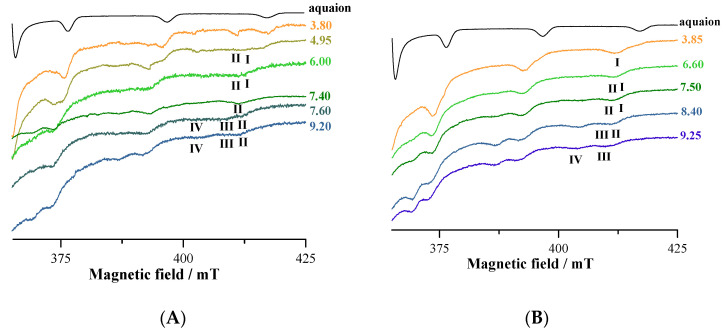
High-field region of the anisotropic X-band EPR spectra recorded on frozen solutions (120 K) of V^IV^O^2+^-L9 system at 1:1 (**A**) and 1:2 (**B**) molar ratio at V^IV^O^2+^ concentration 2 mM at different pH.

The EPR spectra of V^IV^O^2+^-L9 system at 1:1 and 1:2 molar ratio at increasing pH show the progressive formation of four species indicated with **I**–**IV** (Figure 5). The pH dependence is similar to that indicated in the distribution curves in Figure 2.

Species **I** is attributed to the mono-chelated complex with ‘KA-like’ coordination (CO, O^−^); H_2_O; H_2_O; H_2_O, [V^IV^OLH_2_]^2+^complex or [(V^IV^O)_2_L_2_H_3_]^3+^ (with NH^+^ and OH protonated), confirming potentiometric and spectrophotometric data. Spin Hamiltonian parameters are *g*_z_ = 1.938 and *A*_z_ = 177.0 × 10^−4^ cm^−1^, in agreement with the data in the literature [30,56,58]. This could correspond also to [(V^IV^O)_2_L_2_H_2_]^2+^ (with one NH^+^ and OH protonated). The involvement of a second KA in the coordination is observed in species **II**, which has *g*_z_ = 1.943 and *A*_z_ = 170.1 × 10^−4^ cm^−1^. This should correspond to the binuclear species [(V^IV^O)_2_L_2_H_2_]^2+^, where the two ligands act as a bridge between each V^IV^O^2+^ center and bind the metal through ‘KA-like’ coordination (CO, O^−^); (CO, O^−^); H_2_O in an equatorial–equatorial and in an equatorial–axial coordination [56,57,58].

The mono-hydroxido complex with ‘KA-like’ coordination (CO, O^−^); (CO, O^−^); OH^−^ is formed at higher pH values (species **III**, *g*_z_ = 1.945 and *A*_z_ = 168.1 × 10^−4^ cm^−1^) and its composition is [(V^IV^O)_2_L_2_H]^+^. The simultaneous deprotonation of more than one water ligand to give OH^−^ complexes is not favored and rarely was observed in the literature; when this occurs, polynuclear species are formed. The resonances of the species **IV**, formed at pH > 7, may be assigned to the mono-hydroxido complex with (O^−^, N, O^−^); OH^−^ coordination. This species with the ligand in the fully deprotonation form is observed at basic pH for tridentate ligands with two phenolato-O^−^ and one amino/aromatic-nitrogen [63,64].

The large broad signals observed from pH 3 to 4 (when the formation of the binuclear species starts) could be due to the presence of the dimers and a small amount of EPR-active hydrolytic or polynuclear V^IV^O complexes [65]. Notably, ESI-MS shows the presence in solution of species with such features, with formula [(V^V^O_2_)(V^IV^O)_2_(L9)_3_+*x*H]^y+^, [(V^V^_2_O_3_)(V^IV^O)_2_(L9)_4_+*x*H]^y+^ and [(V^V^_2_O_3_)(V^IV^O)_2_(L9)_4_+H_2_O+*x*H]^y+^ (Table 3), which could escape, at least to a first approximation, to the spectrophometric/potentiometric titrations.

**Figure 6:**
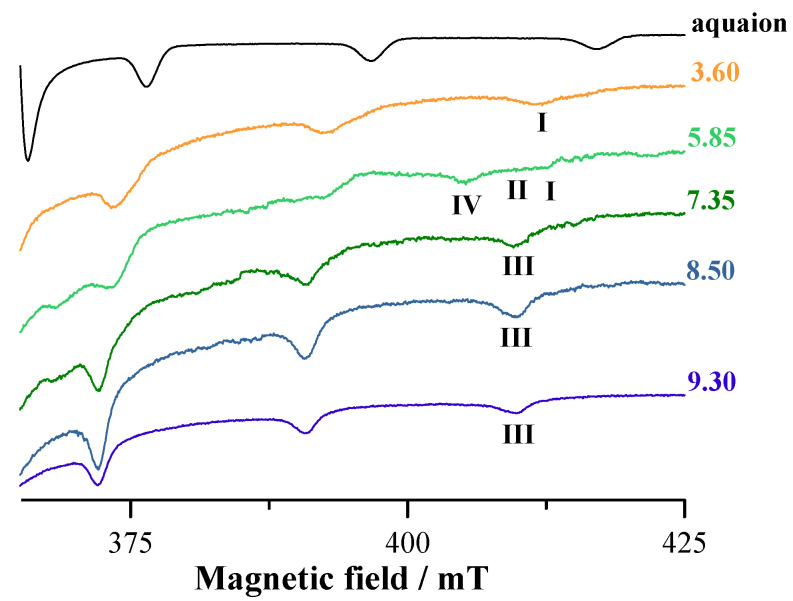
High-field region of the anisotropic X-band EPR spectra recorded on frozen solutions (120 K) of V^IV^O^2+^-L4 at 1:2 molar ratio and V^IV^O^2+^ concentration 2 mM at different pH.

Similar comments can be made regarding the systems with L4 whose EPR spectra collected in the V^IV^O^2+^-L4 system at 1:2 molar ratio as a function of pH are shown in Figure 6.

The behavior of the systems with ratio 1:1 and 1:2 is comparable, but resolution of the EPR spectra improves when the ratio metal to ligand is increased to 1:2. The species indicated by **I** is the mono-chelated complex with ‘KA-like’ coordination (CO, O^−^); H_2_O; H_2_O; H_2_O and formula [V^IV^OLH_2_]^2+^ (with one NH^+^ and OH protonated). Spin Hamiltonian parameters are *g*_z_ = 1.939 and *A*_z_ = 176.6 × 10^−4^ cm^−1^ [56,58]. Species **II** is the bis-chelated complex with KA donor set (CO, O^−^); (CO, O^−^); H_2_O and with EPR parameters *g*_z_ = 1.943 and *A*_z_ = 170.1 × 10^−4^ cm^−1^; the arrangement of the two KA groups is (equatorial–equatorial) and (equatorial–axial) and corresponds to [(V^IV^O)_2_L_2_H_2_]^2+^ and [(V^IV^O)_2_L_2_H]^+^ [56,58]. Species **III** is the mono-hydroxido complex [(V^IV^O)_2_L_2_] with ‘KA-like’ coordination (CO, O^−^); (CO, O^−^); OH^−^ and spin Hamiltonian parameters are *g*_z_ = 1.944 and *A*_z_ = 168.0 × 10^−4^ cm^−1^ [53,55]. The spin Hamiltonian parameters for the species **IV**, observed at pH around 5, are unusual and could be attributed to a non-oxido V^IV^ complexes with compositions VL_2_·H_2_O (i.e., VL_2_H_2_), present in small amounts in solution. In this species, the ligand is in the fully deprotonated form and binds V^IV^ with (O^−^, N, O^−^), similarly to other ligands with two phenolato-O^−^ and one amino/aromatic-nitrogen [63,64,66].

### 3.6. NMR Experiments

**Figure 7:**
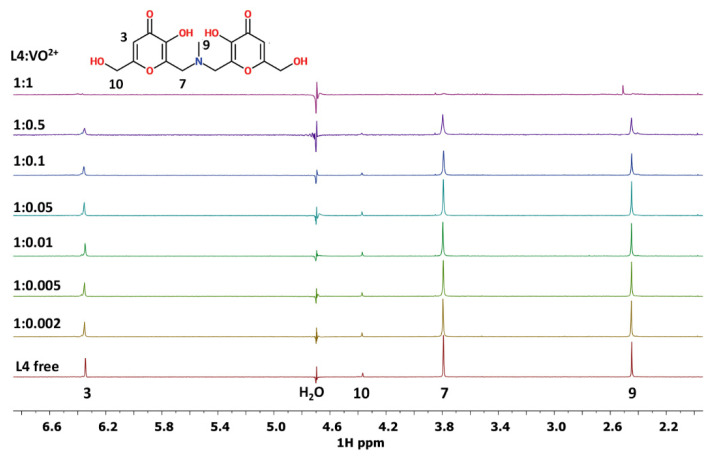
1D ^1^H NMR spectra of L4-V^IV^O^2+^ system in D_2_O at different molar ratios.

The 1D spectra of L4 and L9 ligands in MeOD solution are reported in Figures S21 and S22, respectively. The assignments of the free ligands in D_2_O solution were previously reported [31,34]. The spectra of L4-V^IV^O^2+^ system in water are reported in Figure 7 at different L4:V^IV^O^2+^ ratios varying from the 1:0.002 to 1:1. From this figure, it is possible to observe that all the proton signals progressively disappear, due to the paramagnetic effect of the V^IV^O^2+^ ion, except the signal corresponding to protons in position 9, which decreases in intensity and undergoes a small shift additionally. The same behavior has been evidenced in ^1^H and in ^1^H-^1^H COSY spectra in MeOD solution (Figures S23 and S24).

Concerning the L9 ligand, the increase of L9:V^IV^O^2+^ molar ratio from 1:0.002 to 1:1, in water solution at pH 7.4, causes a selective decreasing of the signal intensities, involving protons 3, 14 and 7 in particular, and to a lesser extent, in protons 10, 12, 9 and 13. This suggests that the paramagnetic ion is more distant from these atoms than from the previous ones. From HSQC spectra, it is possible to evidence that the less affected atoms are located close to N11.

**Figure 8:**
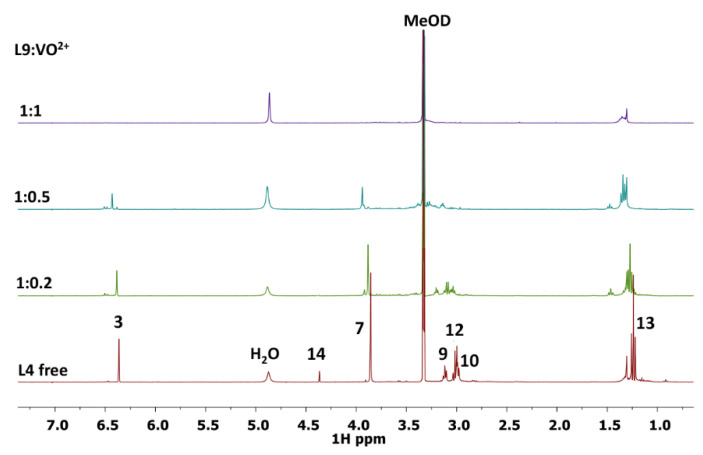
1D ^1^H NMR spectra of L9-V^IV^O^2+^ system in MeOD at different L9:V^IV^O^2+^ ratios.

The same behavior, a decrease of the signal intensity together with a small shift, has been observed in the L9-V^IV^O^2+^ system in MeOD solution, indicating proton 13 and, to a lesser extent, proton 12 as the atoms furthest away from the paramagnetic center (Figure 8).

**Figure 9:**
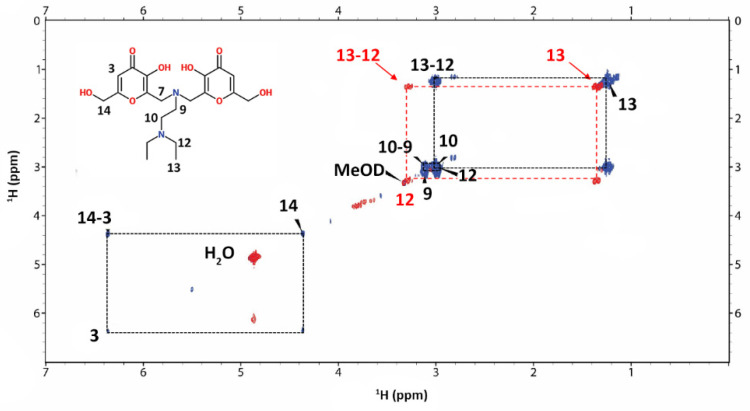
Comparison of 2D ^1^H-^1^H NMR COSY spectra of L9 free (blue) and L9-V^IV^O^2+^ (red) systems in MeOD solution.

In the MeOD COSY spectra (Figure 9) the new correlation between the signals of the protons 12–13 of the L9-V^IV^O^2+^ species appears.

Taking into account the structure of L4 and L9 molecules, it is possible to confirm from NMR results the involvement of oxygen donor atoms in the coordination to V^IV^O^2+^ ion. The occurrence of the signals of proton 9 for L4 ligand and of 12 and 13 for L9 ligand suggests that these protons are the most distant ones from the paramagnetic site. In addition, the variation in the chemical shift for the proton 9 of L4 ligand and for the protons 12 and 13 of L9 ligand indicates that a high symmetry characterizes the molecular structure of the obtained V^IV^O^2+^ complexes.

The results acquired from NMR measurements are in agreement with all the data obtained with the complementary spectrometric and spectroscopic techniques used to characterize the systems V^IV^O^2+^-L4 and V^IV^O^2+^-L9.

### 3.7. DFT Calculations

**Figure 10:**
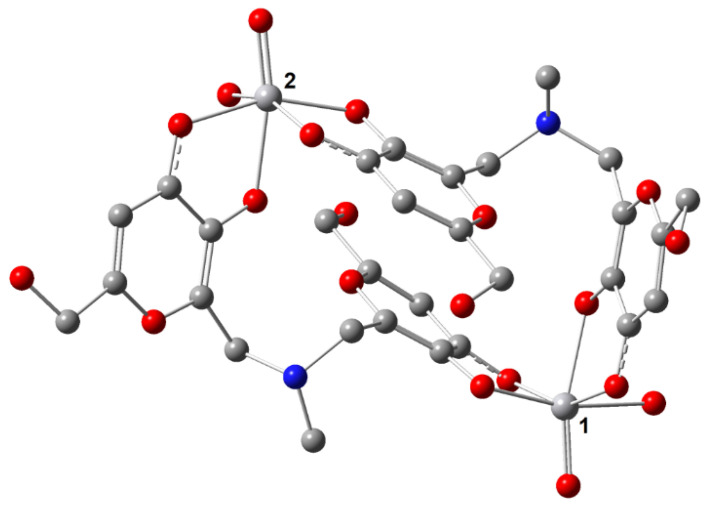
DFT optimized structure of [(V^IV^O)_2_(L4)_2_(H_2_O)_2_]. The V**⋯**V distance is 8.88 Å. The two V^IV^O^2+^ centers are indicated with **1** and **2**. The hydrogen atoms are omitted for clarity.

To characterize the binuclear complexes observed in solution by potentiometry and mass spectrometry, the structure [(V^IV^O)_2_(L4)_2_(H_2_O)_2_], with two water ligands equatorially bound to V^IV^O^2+^, was DFT optimized. Subsequently, the magnetic coupling between the V^IV^O^2+^ centers and *A*_z_(^51^V) of the two metal ions was predicted. In the optimized structure (Figure 10), the distance between the two vanadium atoms is 8.882 Å; the V=O lengths are 1.607 and 1.610 Å, the equatorial V–O(phen) and V–O(keto) are 1.973–1973 Å and in the range 2.022–2.071 Å, respectively, the axial V–O(phen) 2.156 and 2.181 Å and the two V–O(water) bonds 2.156 and 2.187 Å; these data are in line with what was reported for V^IV^O^2+^ species [42]. The equatorial O=V–O angles are between 90.1 and 107.2°, while the axial O=V–O angles are 164.9 and 166.9°. The magnetic interaction between the two V^IV^O^2+^ centers is almost negligible with a very weak ferromagnetic coupling. The predicted value of *J* is 0.11 cm^−1^, in agreement with the EPR data; in fact, the EPR signal experimentally detected (Figure 6) is assignable to a doublet spin state (*S* = ½) that suggests two distinct and almost non-interacting mononuclear V^IV^O units.

In addition, the *A*_z_(^51^V) values of the two V^IV^O^2+^ centers were calculated by the DFT protocol established in the literature, which allows to predict them with a mean absolute percent deviation (MAPD) from the experimental values below 4% and a standard deviation (SD) around 3% [54]. The results are listed in Table 4; the PD values for *A*_z_ are below 1%, in line with the previous results [54].

**Figure 11:**
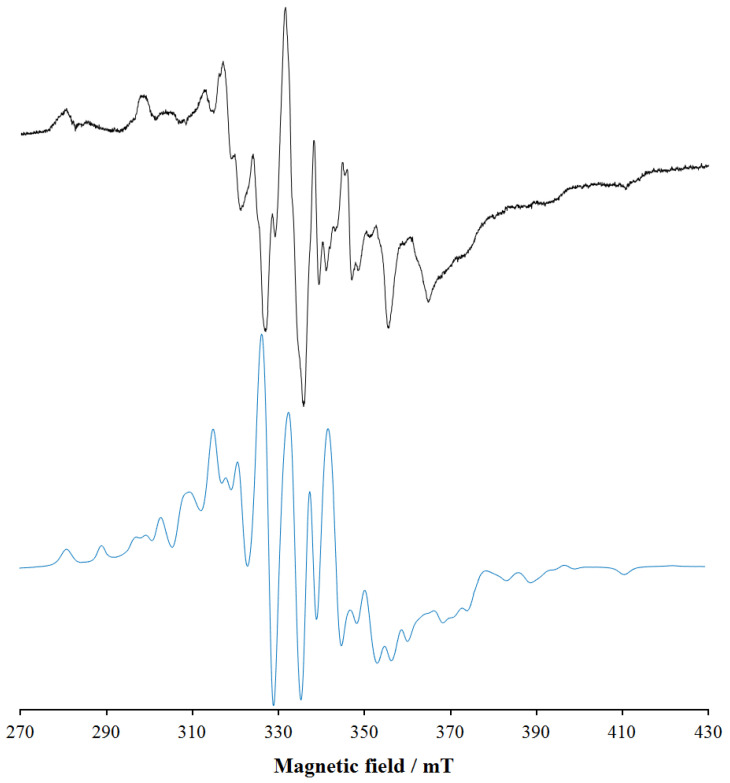
Experimental (top) and simulated (bottom) EPR spectrum of [(V^IV^O)_2_(L4)_2_(H_2_O)_2_] (V^IV^O^2+^-L4 1:1, V^IV^O^2+^ concentration 1 mM, pH 5.1). The spectrum was simulated with EasySpin software, considering two V^IV^O ions with S = 1/2 coupled with *J* = 0.11 cm^−1^. For each V^IV^ center g_x,y,z_ = {1.979, 1.979, 1.943} and A_x,y,z_= {52.5, 52.5, 170.1} × 10^−4^ cm^−1^ were used. Gaussian and Lorentzian broadening were set to 1.4 and 1.4 mT.

The EPR spectrum of [(V^IV^O)_2_(L4)_2_(H_2_O)_2_] was simulated with EasySpin software, using the *J* value calculated by DFT methods and is shown in Figure 11. It can be seen that the simulated spectrum reproduces qualitatively the detected signal, even if the exact agreement cannot be obtained due to the lack of knowledge of the exact experimental value of *J*. The simulations agree well with the data in the literature for V^IV^O dinuclear complexes with comparable *J* [67]. Therefore, on the basis of this result, the broad and unresolved signals observed from pH 3–4 to pH 9–10 in the EPR spectra (see Figure 5 and Figure 6) could be attributed to the presence in solution of V^IV^O dimers with *J* close to 0.1 cm^−1^.

Overall, the data confirm the potentiometric, ESI-MS and EPR results and suggest that in the dimer [(V^IV^O)_2_(L4)_2_(H_2_O)_2_] and [(V^IV^O)_2_(L9)_2_(H_2_O)_2_] the coordination around V^IV^ is octahedral with an (equatorial–equatorial) and an (equatorial–axial) arrangement of the two ligands, as demonstrated for mononuclear complexes formed by KA [58] and maltol [56,58,68].

## 4. Conclusions

The joined use of complementary techniques, such as potentiometry, spectrophotometry, mass spectrometry, EPR and NMR spectroscopy, and DFT calculations has led to the definition of the complexation scheme of V^IV^O^2+^ ion in aqueous solution with the two L4 and L9 kojic acid derivatives. At low pH values, a simple 1:1 complex is formed, with V^IV^O^2+^ coordinated by one of the two KA moieties. Increasing the pH, both ligands form a binuclear complex, in which two KA units from two different ligands coordinate each V^IV^O^2+^ ion.

These binuclear species are very stable at physiological pH, as the pV^IV^O^2+^ values of 12.9 and 11.1 with L4 and L9, respectively, show. The tendency of both the ligands to form binuclear complexes with oxidovanadium(IV) can be explained after careful examination of molecular models. If the two KA moieties of the same ligand molecule were bound simultaneously to only one V^IV^O^2+^ ion, the resulting structure would be very distorted and strained, with the two C=O groups forced to coordinate in the equatorial plane while none of the two C–O^−^ donors could coordinate the metal in the third equatorial position.

As a perspective, these binuclear complexes will be tested as potential insulin-enhancing and cytotoxic agents. Due to their high thermodynamic stability, they could enter intact into the cytosol without interaction with the blood and cellular proteins, which would result in a significant inhibition of the biological and pharmacological activity.

## Figures and Tables

**Table 1:** Protonation constants of L4 and L9 ligands evaluated from potentiometric titration at 25 °C and 0.1 NaCl ionic strength. L indicates the completely deprotonated form of the L4 and L9 ligands.

	L4		L9	
Species	log β	log K	log β	log K
[LH]^−^	9.19 (3)	9.19 ^a^	10.81 (1)	10.81
LH_2_	16.70 (3)	7.51 ^a^	19.04 (2)	8.23 ^a^
[LH_3_]^+^	21.08 (5)	4.38	25.99 (2)	6.95 ^a^
[LH_4_]^2+^			26.50 (4)	0.51

^a^ Protonation constants related to KA moieties.

**Table 2:** Complex formation constants of V^IV^O^2+^ with L4 and L9 evaluated from combined potentiometric-UV titrations at 25 °C, 0.1 M NaCl ionic strength.

	L4		L9	
Species	log β	pK	log β	pK
[V^IV^OLH_2_]^2+^	22.08 (1)		26.03 (3)	
[(V^IV^O)_2_L_2_H_3_]^3+^	–		52.73 (4)	4.41
[(V^IV^O)_2_L_2_H_2_]^2+^	41.63 (2)	4.63	48.32 (2)	7.30
[(V^IV^O)_2_L_2_H]^+^	37.00 (1)	7.24	41.02 (3)	
[(V^IV^O)_2_L_2_]	29.76 (1)		–	
pV^IV^O^2+^	12.9		11.1	

**Table 3:** Species identified in the ESI-MS spectra of the systems V^IV^O^2+^-L4 and V^IV^O^2+^-L9.

Species	Composition	*m/z* (exptl) ^a^	*m/z* (calcd) ^a^	Deviation (ppm) ^b^
[(V^IV^O)_2_(L4)_2_+2H]^2+^	C_30_H_32_N_2_O_18_V_2_	405.02573	405.02592	−0.5
[(V^IV^O)_2_(L4)_2_+H+Na]^2+^	C_30_H_31_N_2_O_18_V_2_Na	416.01658	416.01689	−0.7
[(V^IV^O)_2_(L4)_2_+H]^+^	C_30_H_31_N_2_O_18_V_2_	809.04406	809.04457	−0.6
[(V^IV^O)_2_(L4)_2_+Na]^+^	C_30_H_30_N_2_O_18_V_2_Na	831.02600	831.02651	−0.6
[(V^IV^O)_2_(L4)_2_+K]^+^	C_30_H_30_N_2_O_18_V_2_K	846.99983	847.00045	−0.7
[(V^IV^O)_2_(L9)_2_+2H]^2+^	C_40_H_54_O_18_N_4_V_2_	490.11474	490.11507	−0.7
[(V^IV^O)_2_(L9)_2_+H]^+ c^	C_40_H_53_O_18_N_4_V_2_	979.22286	979.22235	0.5
[(V^V^O_2_)(V^IV^O)_2_(L9)_3_+4H]^3+ d^	C_60_H_82_O_28_N_6_V_3_	495.78245	495.78265	−0.4
[(V^V^O_2_)(V^IV^O)_2_(L9)_3_+3H]^2+ d^	C_60_H_81_O_28_N_6_V_3_	743.17012	743.17034	−0.3
[(V^V^_2_O_3_)(V^IV^O)_2_(L9)_4_+3H]^3+ d^	C_80_H_107_O_37_N_8_V_4_	658.48251	658.48264	−0.2
[(V^V^_2_O_3_)(V^IV^O)_2_(L9)_4_+H_2_O+3H]^3+ d^	C_80_H_109_O_38_N_8_V_4_	664.48597	664.48616	−0.3
[(V^V^_2_O_3_)(V^IV^O)_2_(L9)_4_+H_2_O+2H+Na]^3+ d^	C_80_H_108_O_38_N_8_V_4_Na	671.81325	671.81348	−0.3
[(V^V^_2_O_3_)(V^IV^O)_2_(L9)_4_+H_2_O+2H]^2+ d^	C_80_H_108_O_38_N_8_V_4_	966.22516	966.22560	−0.5

^a^ Experimental and calculated *m/z* values refer to the monoisotopic peak with the highest intensity. ^b^ Error in ppm respect to the experimental value, calculated as 10^6^ × [Experimental (*m/z*)—Calculated (*m/z*)]/Calculated (*m/z*). ^c^ Species detected only in the spectra recorded in MeOH. ^d^ Species detected only in the spectra recorded in H_2_O.

**Table 4:** Experimental and calculated hyperfine coupling constants for [(V^IV^O)_2_(L4)_2_(H_2_O)_2_].

V^IV^O^2+^ Center	*A*_x_^calcd^(^51^V) ^b^	*A*_y_^calcd^(^51^V) ^b^	*A*_z_^calcd^(^51^V) ^b^	*A*_z_^exptl^(^51^V)	PD(*A*_z_) ^a^
**1**	−71.0	−65.5	−168.7	−170.1	−0.8
**2**	−72.1	−69.0	−171.2	−170.1	0.7

^a^ Hyperfine coupling constants reported in 10^−4^ cm^−1^ units. ^b^ Percent deviation of the DFT calculated parameter from the experimental value.

## Data Availability

Data is contained within the article and Supplementary Materials.

## Supplementary Materials

The following are available online at https://www.mdpi.com/article/10.3390/ph14101037/s1, Figure S1: ESI-MS/MS(+) spectrum of the signal relative to the [L9+3H]^+^ ion selected in the range of *m/z* = 425.19 ± 0.5, NCE = 10, recorded on the system V^IV^O^2+^-L9 at 1:1 V ^IV^O^2+^: ligand molar ratio at ligand concentration 5 µM (MeOH); Figure S2: ESI-MS/MS(+) spectrum of the signal relative to the [L4+H]^+^ ion selected in the range of *m/z* = 340.10 ± 0.5, NCE = 10, recorded on the system V^IV^O^2+^-L4 at 1:1 V^IV^O^2+^: ligand molar ratio with [L4] = 50 µM; Figure S3: Representative spectra of UV titration of L4 ligand at ligand concentration 3 × 10^−4^ M. Top: beginning of titration at λ = 274 nm and pH 3.89. Bottom: end of titration at λ = 326 nm and pH = 10.06; Figure S4: Molar absorptivity of L4 ligand; Figure S5: Representative spectra of UV titration of L9 ligand at ligand concentration 3 × 10^−4^ M. Top: beginning of titration at λ = 276 nm and pH 4.87. Bottom: end of titration at λ = 330 nm and pH = 10.99; Figure S6: Molar absorptivity of L9 ligand; Figure S7: UV titration of V^IV^O^2+^-L4 at 1:1 V^IV^O^2+^: ligand molar ratio at ligand concentration 3 × 10^−4^ M. HypSpec screenshot. Top: λ = 272 nm, pH 3.33. Middle: λ = 322 nm, pH 7.00. Bottom: λ = 322 nm, pH 9.98; Figure S8: Molar absorptivity of V^IV^O^2+^-L4 at 1:1 V^IV^O^2+^: ligand molar ratio at ligand concentration 3 × 10^−4^ M; Figure S9: UV titration of V^IV^O^2+^-L4 at 1:2 V^IV^O^2+^: ligand molar ratio at ligand concentration 3 × 10^−4^ M: HypSpec screenshot. λ = 320 nm, pH 9.00; Figure S10: UV titration of V^IV^O^2+^-L4 at 1:4 V^IV^O^2+^: ligand molar ratio at ligand concentration 3 × 10^−4^ M: HypSpec screenshot. λ = 322 nm, pH 8.71; Figure S11: UV titration of V^IV^O^2+^-L9 at 1:1 V^IV^O^2+^: ligand molar ratio at ligand concentration 3 × 10^−4^ M: HypSpec screenshot. Top: λ = 276 nm, pH 2.51. Middle: λ = 276 nm, pH 7.00. Bottom: λ = 322 nm, pH 9.98.; Figure S12: Molar absorptivity of V^IV^O^2+^-L9 at 1:1 V^IV^O^2+^: ligand molar ratio at ligand concentration 3 × 10^−4^ M.; Figure S13: Molar absorptivity of V^IV^O^2+^-L9 at 1:1 V^IV^O^2+^: ligand molar ratio at ligand concentration 3 × 10^−4^ M; Figure S14: UV titration of V^IV^O^2+^-L9 at 1:4 V^IV^O^2+^: ligand molar ratio at ligand concentration 3 × 10^−4^ M: HypSpec screenshot. λ = 364 nm, pH 8.38.; Figure S15: Experimental (top) and calculated (bottom) isotopic pattern for the peak of [(V^IV^O)_2_(L4)^2+^2H]^2+^ detected in the ESI-MS(+) spectrum of the system V^IV^O^2+^-L4 at 1:1 molar ratio (LC-MS H_2_O, ligand concentration 50 µM); Figure S16: Experimental (top) and calculated (bottom) isotopic pattern for the peak of [(V^IV^O)_2_(L4)^2+^H]^+^ detected in the ESI-MS(+) spectrum of the system V^IV^O^2+^-L4 at 1:1 molar ratio (LC-MS H_2_O, ligand concentration 50 µM).; Figure S17: ESI-MS(+) spectrum recorded on the system V^IV^O^2+^-L9 at 1:1 molar ratio (LC-MS MeOH, ligand concentration 5 µM); Figure S18: Experimental (top) and calculated (bottom) isotopic pattern for the peak of [(V^IV^O)_2_(L9)^2+^2H]^2+^ detected in the ESI-MS(+) spectrum of the system VIVO^2+^-L9 at 1:1 molar ratio (LC-MS MeOH, ligand concentration 5 µM); Figure S19: Experimental (top) and calculated (bottom) isotopic pattern for the peak of [(V^IV^O)_2_(L9)^2+^H]^+^ detected in the ESI-MS(+) spectrum of the system V^IV^O^2+^-L9 at 1:1 molar ratio (LC-MS MeOH, ligand concentration 5 µM); Figure S20: Experimental (top) and calculated (bottom) isotopic pattern for the peak of [(V^V^_2_O_3_)(V^IV^O_2_)_2_(L9)^4+^3H]^3+^ detected in the ESI-MS(+) spectrum of the system V^IV^O^2+^-L9 at 1:1 molar ratio (LC-MS H_2_O, ligand concentration 50 µM); Figure S21: 1D 1HNMR spectra of free ligands L4 (bottom) and L9 (top) in MeOD; Figure S22: NMR HSQC of L4 and L9 ligands in MeOD; Figure S23: 1D 1HNMR spectra of L4-V^IV^O^2+^ system in MeOD at different L4:V^IV^O^2+^ ratios; Figure S24: Comparison of 2D 1H-1H NMR COSY spectra of L4 free (blue) and L4-V^IV^O^2+^ (red) systems in MeOD solution; Table S1: Species identified in the ESI-MS spectra of the L4 and L9 ligands.
