# Supplementary material for: Thermodynamic Study of Oxidovanadium(IV) with Kojic Acid Derivatives: A Multi-Technique Approach
Source: Pharmaceuticals (Basel). 2021 Oct 12;14(10):1037. doi: 10.3390/ph14101037 (PMC8541509; doi:10.3390/ph14101037)
Supplement: Supplementary file 1 [file pharmaceuticals-14-01037-s001.zip › pharmaceuticals-1386246-supplementary.pdf]

pharmaceuticals

Supplementary Materials

Thermodynamic Study of Oxidovanadium(IV) with Kojic Acid Derivatives: A Multi-Technique Approach

Rosita Cappai [1,*], Guido Crisponi [1], Daniele Sanna [2], Valeria Ugone [2], Andrea Melchior [3], Eugenio Garribba [4], Massimiliano Peana [4], Maria Antonietta Zoroddu [4] and Valeria Marina Nurchi [1,*]

[1] Dipartimento di Scienze della Vita e dell'Ambiente, Università di Cagliari; 09042 Monserrato-Cagliari, Italy; crisponi@unica.it (G.C.)
[2] Istituto di Chimica Biomolecolare, Consiglio Nazionale delle Ricerche, Trav. La Crucca 3, 07100 Sassari, Italy; daniele.sanna@cnr.it (D.S.); valeria.ugone@cnr.it (V.U.)
[3] DPIA, Laboratorio di Scienze e Tecnologie Chimiche, Università di Udine, Via del Cotonificio 108, 33100 Udine, Italy; andrea.melchior@uniud.it
[4] Dipartimento di Chimica e Farmacia, Università di Sassari, via Vienna 2, 07100 Sassari, Italy; garribba@uniss.it (E.G.); peana@uniss.it (M.P.); zoroddu@uniss.it (M.A.Z.)
* Correspondence: cappai@unica.it (R.C.); nurchi@unica.it (V.M.N.)

Citation: Cappai, R.; Crisponi, G.; Sanna, D.; Ugone, V.; Melchior, A.; Garribba, E.; Peana, M.; Zoroddu, M.A.; Nurchi, V.M. Thermodynamic Study of Oxidovanadium(IV) with Kojic Acid Derivatives: A Multi-Technique Approach. *Pharmaceuticals* **2021**, *14*, x. https://doi.org/10.3390/xxxxx

Academic Editor: Micheline Draye and Mary J. Meegan

Received: 2 September 2021
Accepted: 7 October 2021
Published: date

Publisher's Note: MDPI stays neutral with regard to jurisdictional claims in published maps and institutional affiliations.

Copyright: © 2021 by the authors. Submitted for possible open access publication under the terms and conditions of the Creative Commons Attribution (CC BY) license (https://creativecommons.org/licenses/by/4.0/).

Table S1. Species identified in the ESI-MS spectra of the L4 and L9 ligands.

Species	Composition	m/z (exptl) [a]	m/z (calcd) [a]	Deviation (ppm) [b]
$[L4+H]^+$	$C_{15}H_{18}NO_8$	340.10250	340.10269	−0.6
$[L4+Na]^+$	$C_{15}H_{17}NO_8Na$	362.08446	362.08464	−0.5
$[L9+H]^+$	$C_{20}H_{29}O_8N_2$	425.19134	425.19184	−1.2

[a] Experimental and calculated m/z values refer to the monoisotopic peak with the highest intensity. [b] Error in ppm respect to the experimental value, calculated as $10^6 \times$ [Experimental (m/z) – Calculated (m/z)] / Calculated (*m*/*z*).

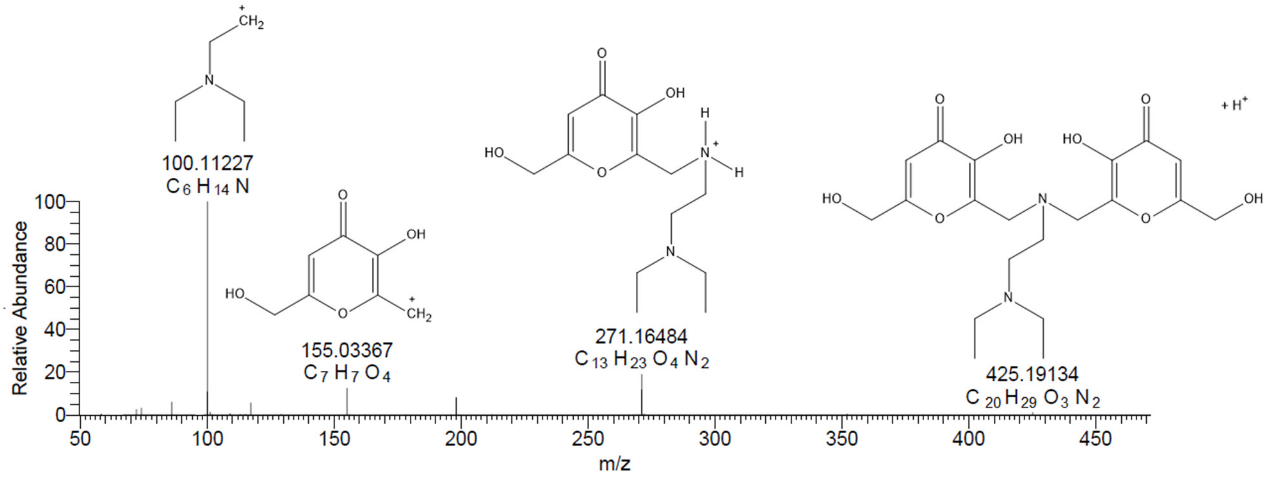

Figure S1. ESI-MS/MS(+) spectrum of the signal relative to the $[L9+3H]^+$ ion selected in the range of *m*/*z* = 425.19 ± 0.5, NCE = 10, recorded on the system $V^{IV}O^{2+}$-L9 at 1:1 $V^{IV}O^{2+}$:ligand molar ratio at ligand concentration 5μM (MeOH).

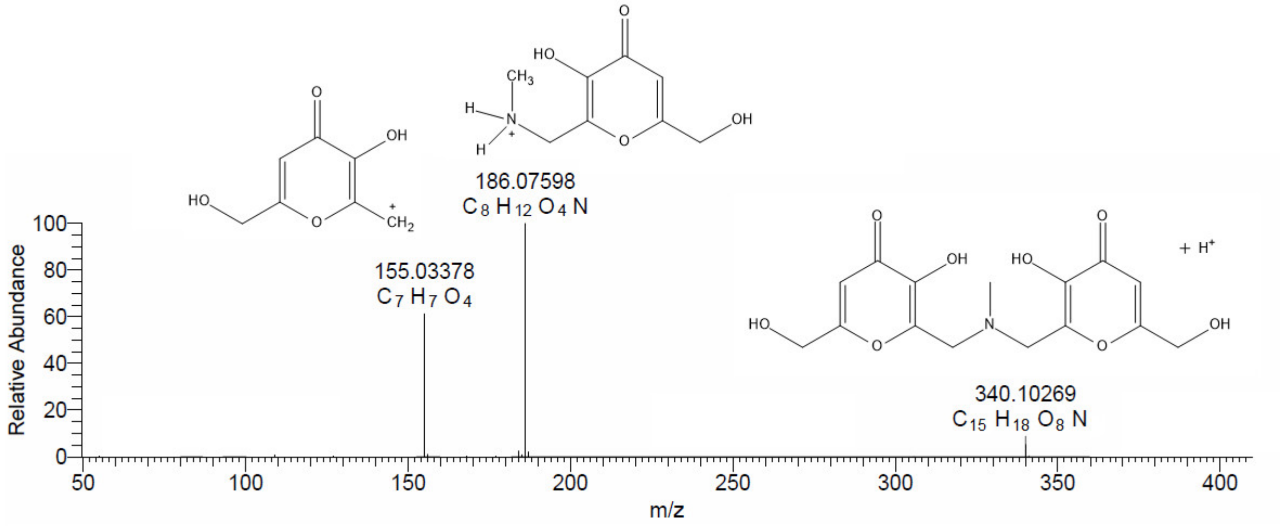

Figure S2. ESI-MS/MS(+) spectrum of the signal relative to the $[L4+H]^+$ ion selected in the range of *m*/*z* = 340.10 ± 0.5, NCE = 10, recorded on the system $V^{IV}O^{2+}$-L4 at 1:1 $V^{IV}O^{2+}$:ligand molar ratio with [L4] = 50 μM.

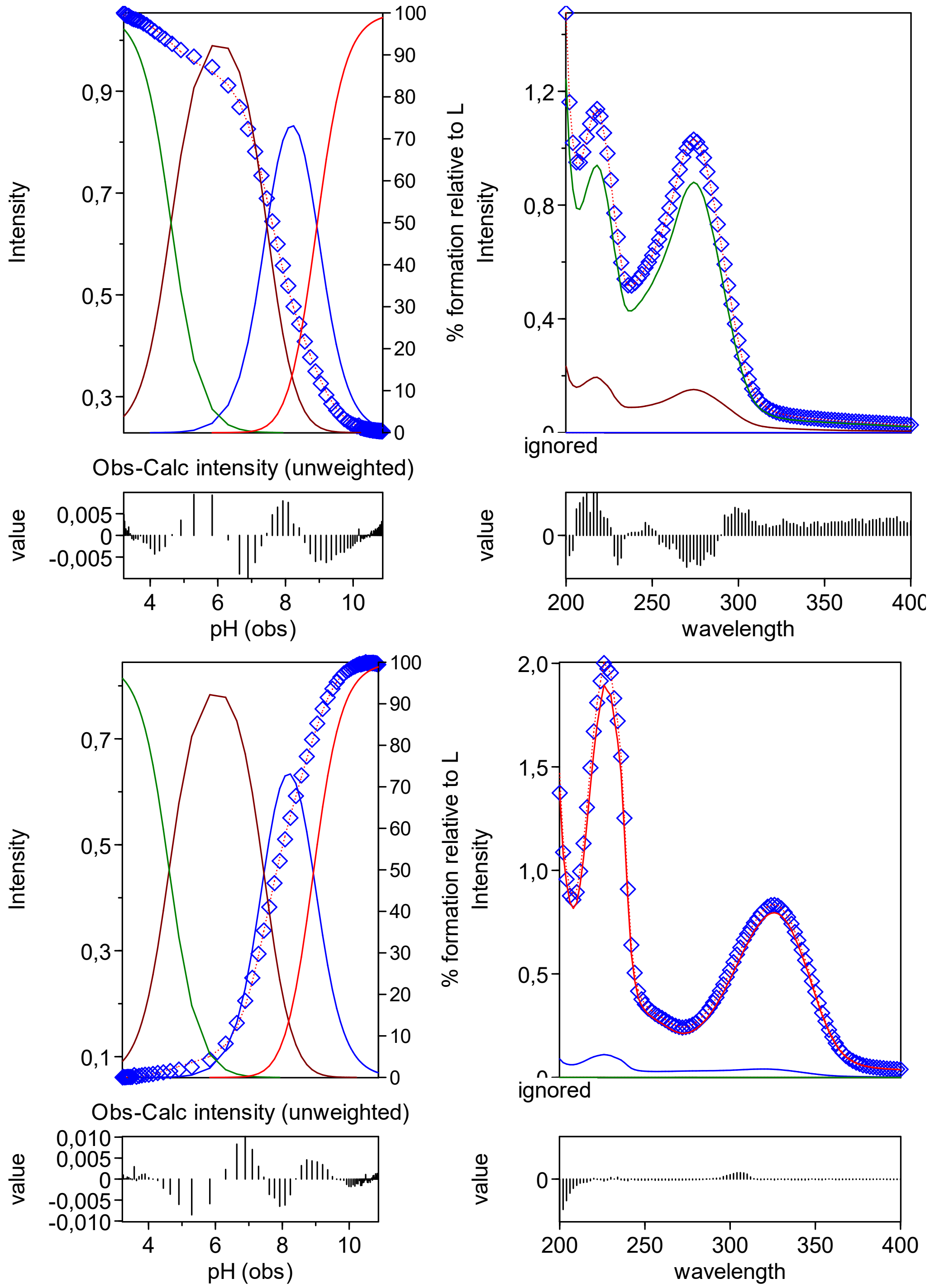

Figure S3. Representative spectra of UV titration of L4 ligand at ligand concentration 3×10^{-4}M. Top: beginning of titration at $\lambda = 274$ nm and pH 3.89. Bottom: end of titration at $\lambda = 326$ nm and pH = 10.06.

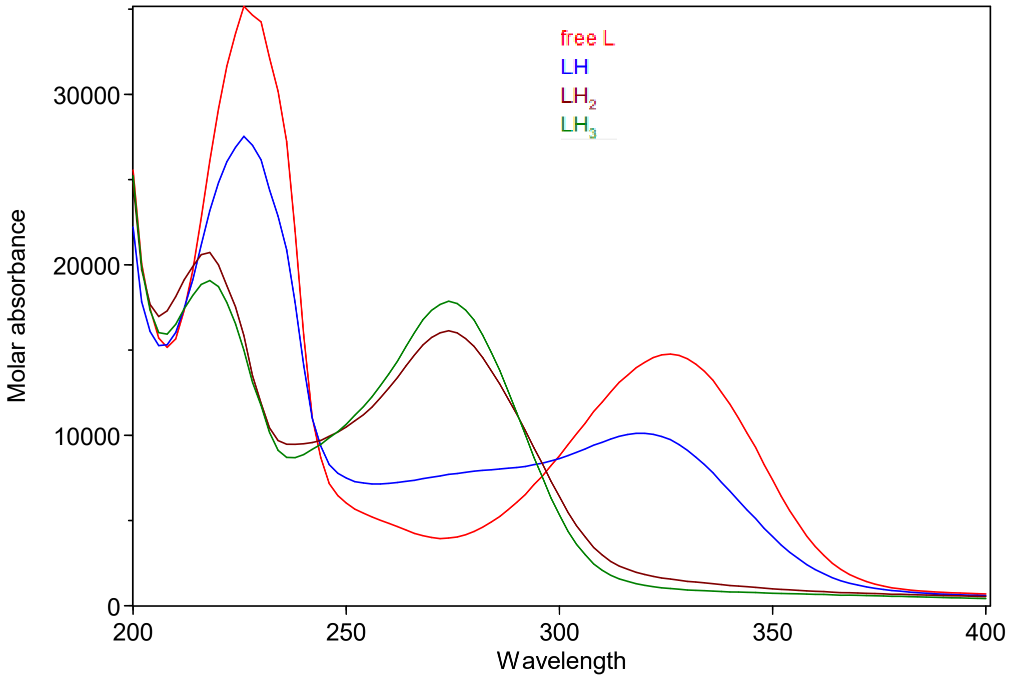

Figure S4. Molar absorptivity of L4 ligand.

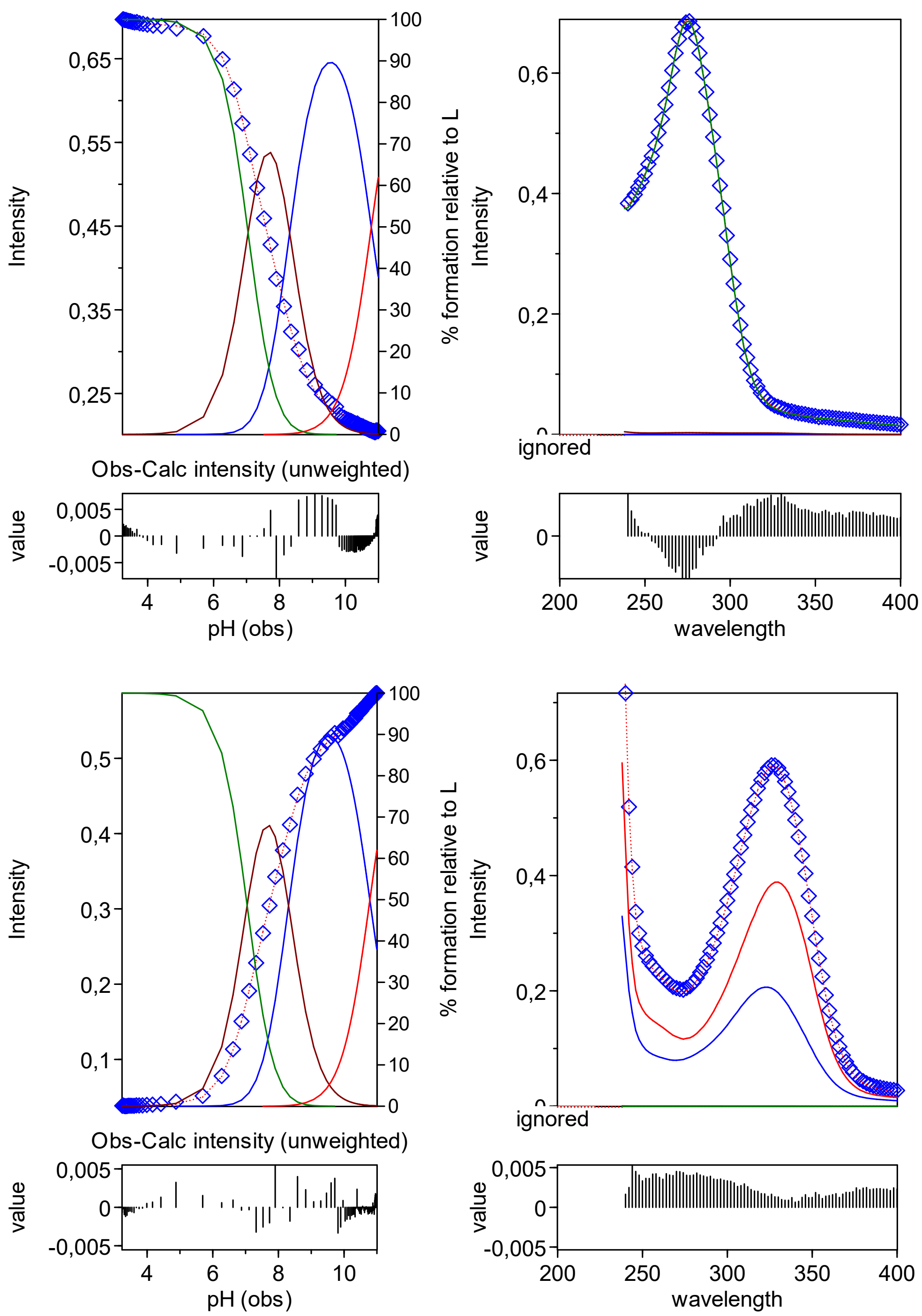

Figure S5. Representative spectra of UV titration of L9 ligand at ligand concentration 3×10^{-4} M. Top: beginning of titration at λ = 276 nm and pH 4.87. Bottom: end of titration at λ = 330 nm and pH = 10.99.

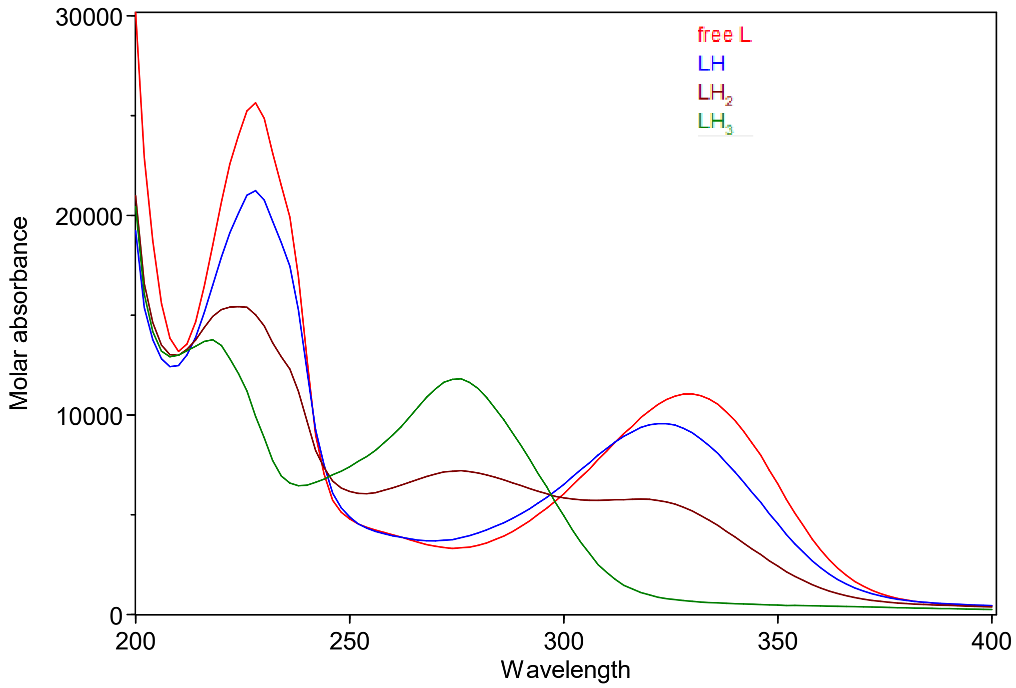

Figure S6. Molar absorptivity of L9 ligand.

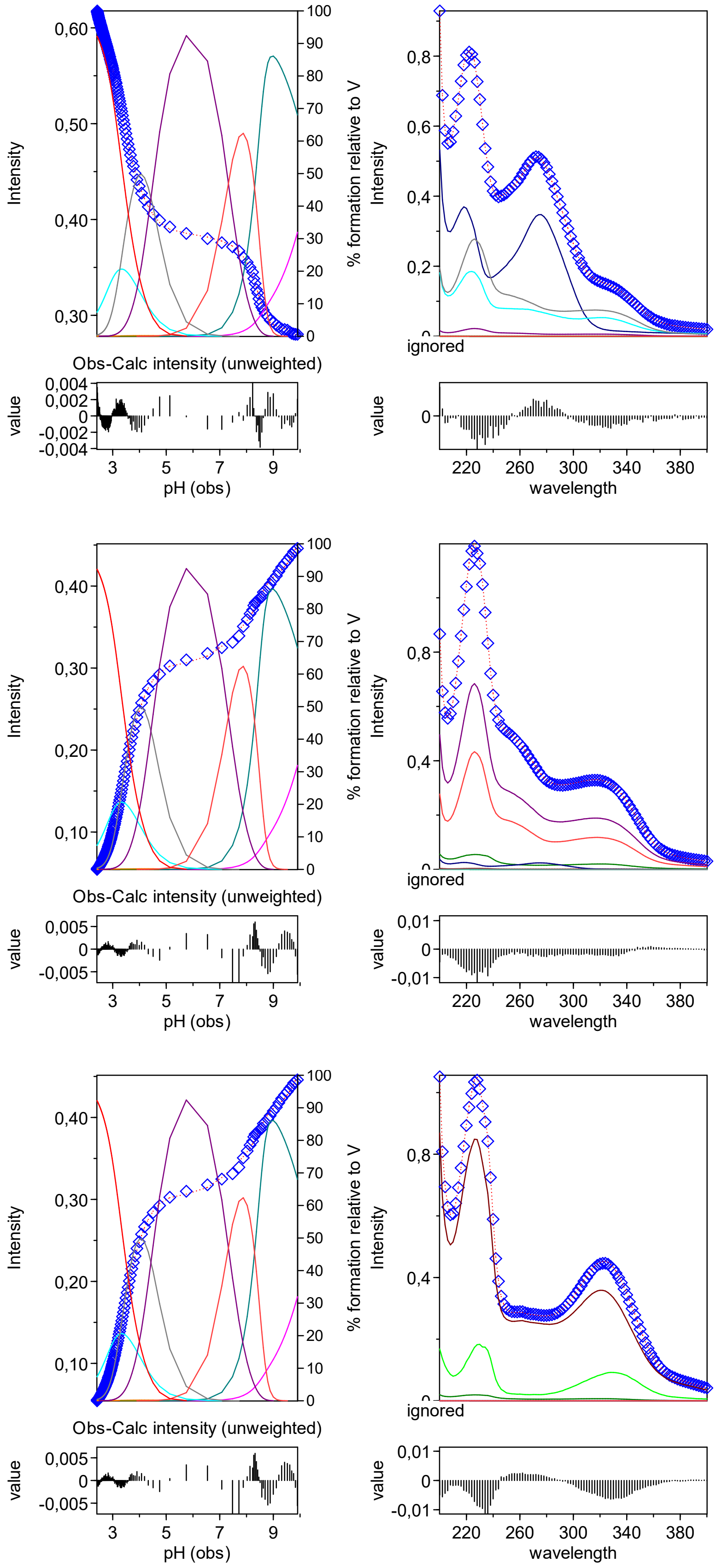

Figure S7. UV titration of $V^{IV}O^{2+}$-L4 at 1:1 $V^{IV}O^{2+}$:ligand molar ratio at ligand concentration 3×10^{-4} M. HypSpec screenshot. Top: λ = 272 nm, pH 3.33. Middle: λ = 322 nm, pH 7.00. Bottom: λ = 322 nm, pH 9.98.

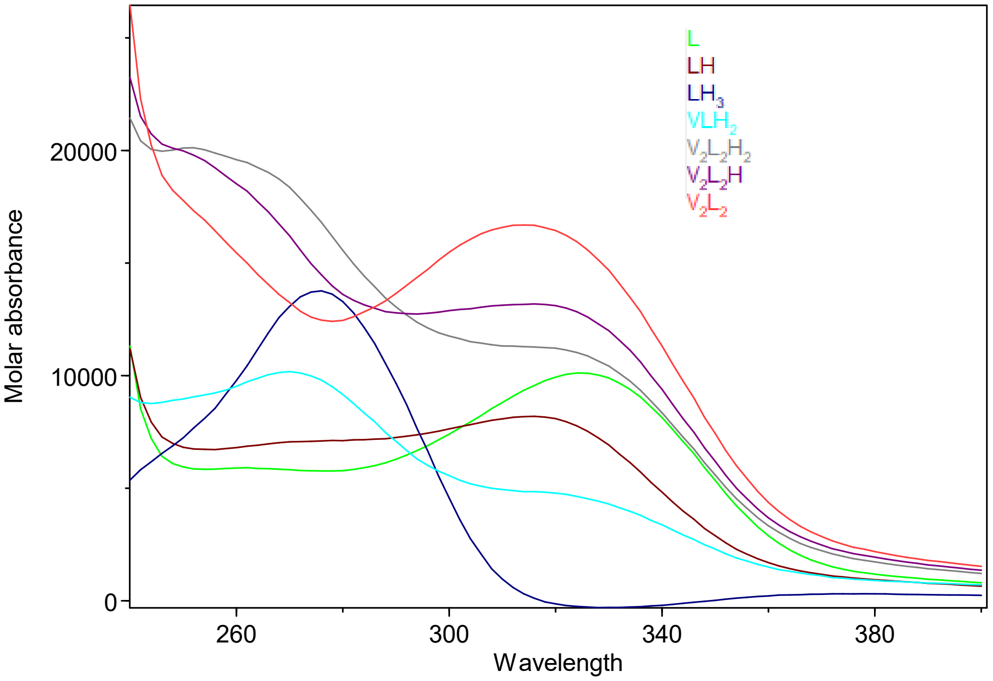

Figure S8. Molar absorptivity of $V^{IV}O^{2+}$-L4 at 1:1 $V^{IV}O^{2+}$:ligand molar ratio at ligand concentration $3{\times}10^{-4}$ M.

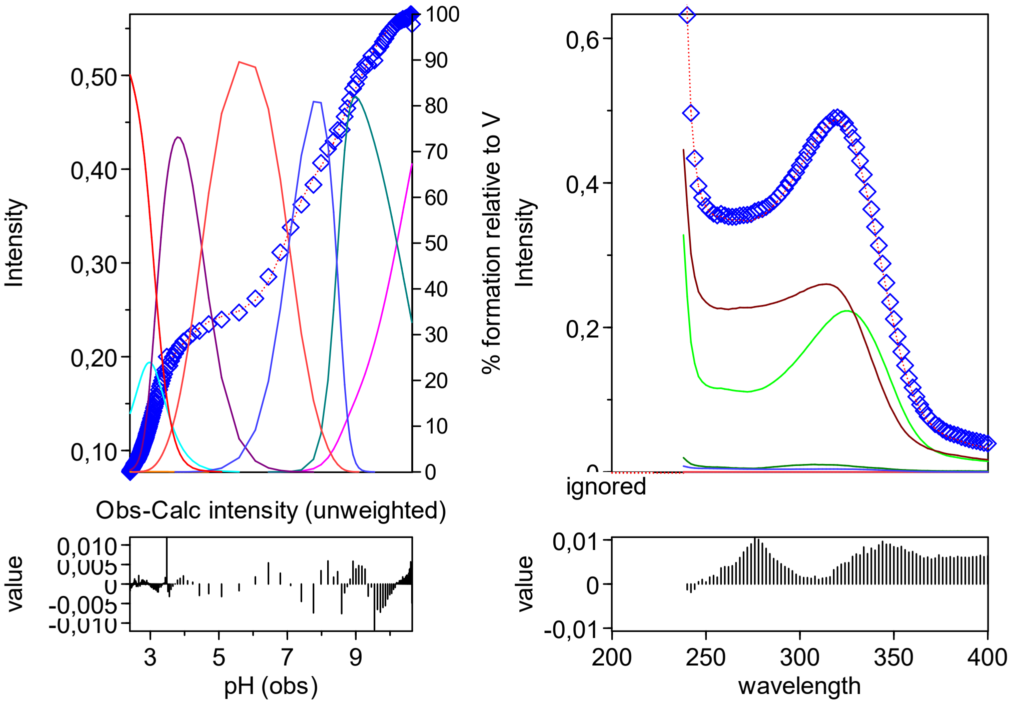

Figure S9. UV titration of $V^{IV}O^{2+}$-L4 at 1:2 $V^{IV}O^{2+}$:ligand molar ratio at ligand concentration $3{\times}10^{-4}$M: HypSpec screenshot. λ = 320 nm, pH 9.00.

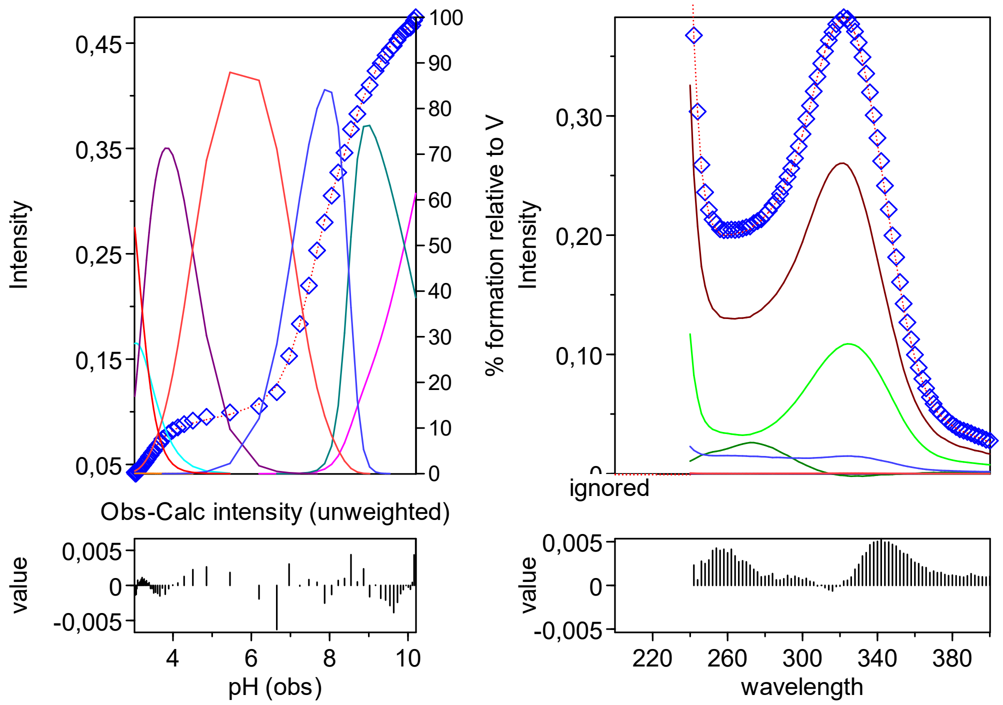

Figure S10. UV titration of $V^{IV}O^{2+}$-L4 at 1:4 $V^{IV}O^{2+}$:ligand molar ratio at ligand concentration 3×10^{-4} M: HypSpec screenshot. λ = 322 nm, pH 8.71.

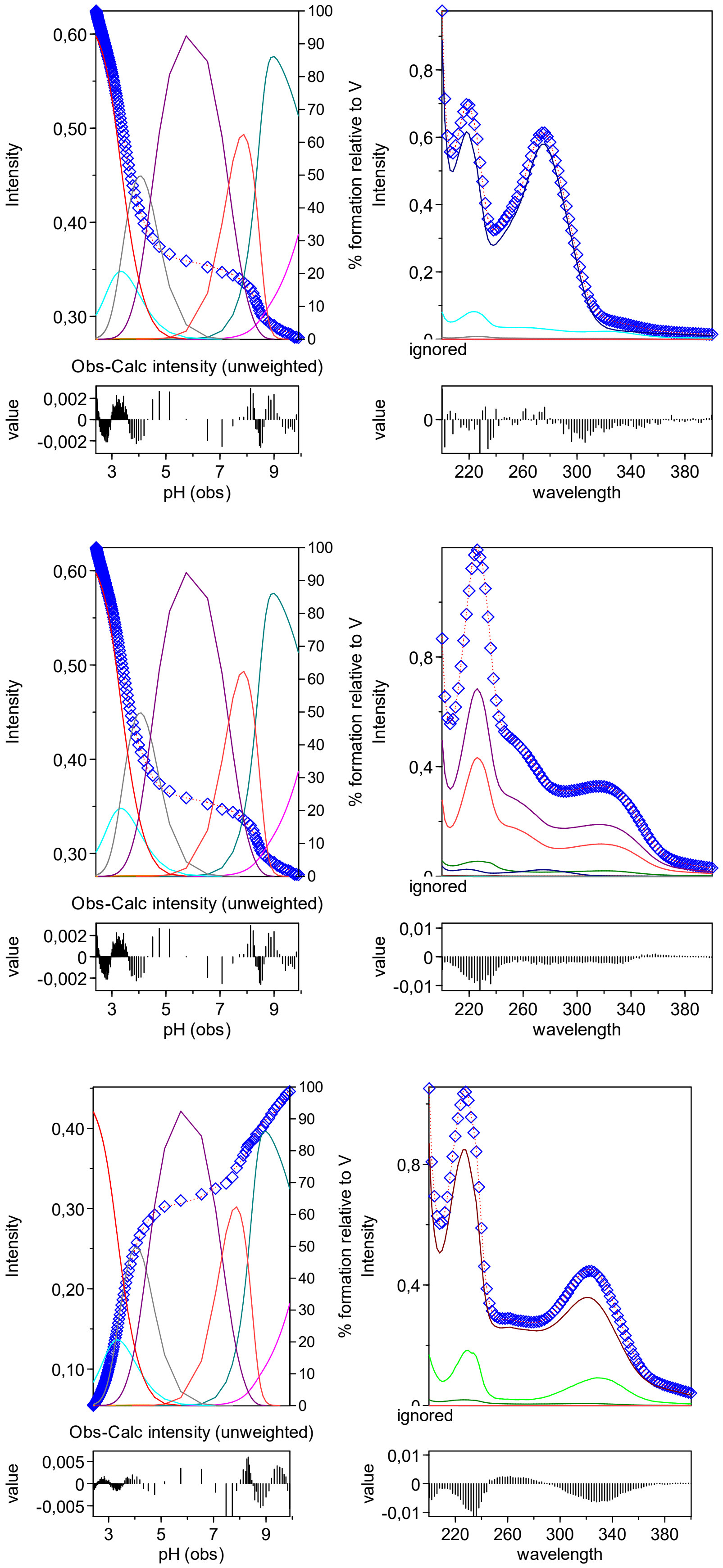

Figure S11. UV titration of $V^{IV}O^{2+}$-L9 at 1:1 $V^{IV}O^{2+}$:ligand molar ratio at ligand concentration $3{\times}10^{-4}$M: HypSpec screenshot. Top: λ = 276 nm, pH 2.51. Middle: λ = 276 nm, pH 7.00. Bottom: λ = 322 nm, pH 9.98.

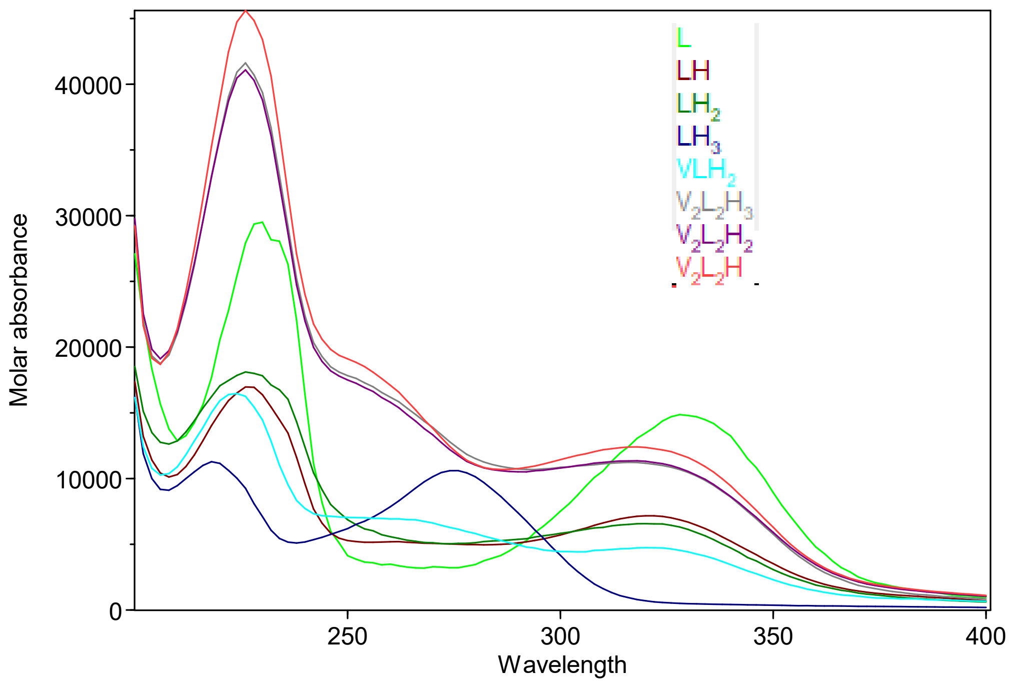

Figure S12. Molar absorptivity of $V^{IV}O^{2+}$-L9 at 1:1 $V^{IV}O^{2+}$:ligand molar ratio at ligand concentration 3×10^{-4} M.

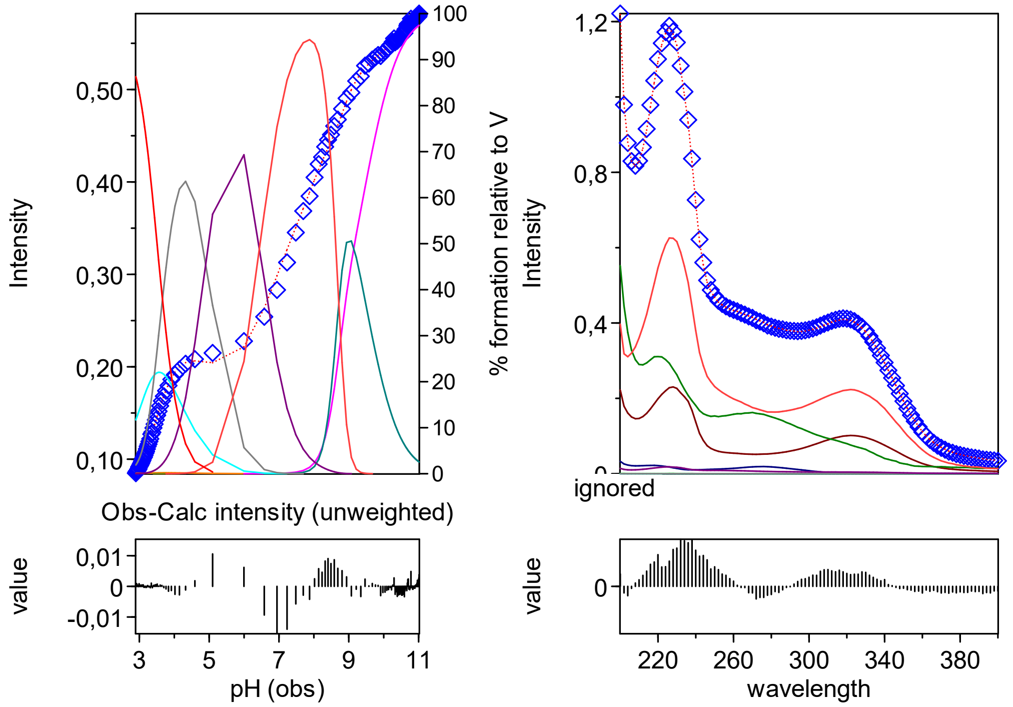

Figure S13. Molar absorptivity of $V^{IV}O^{2+}$-L9 at 1:1 $V^{IV}O^{2+}$:ligand molar ratio at ligand concentration 3×10^{-4} M.

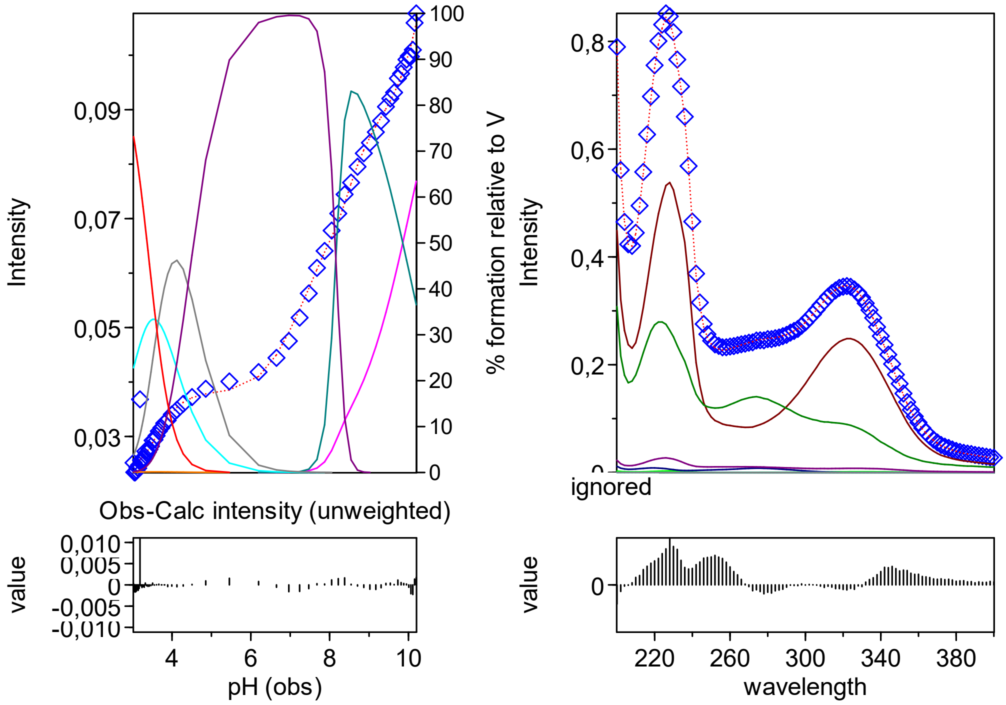

Figure S14. UV titration of $V^{IV}O^{2+}$-L9 at 1:4 $V^{IV}O^{2+}$:ligand molar ratio at ligand concentration 3×10^{-4} M: HypSpec screenshot. λ = 364 nm, pH 8.38.

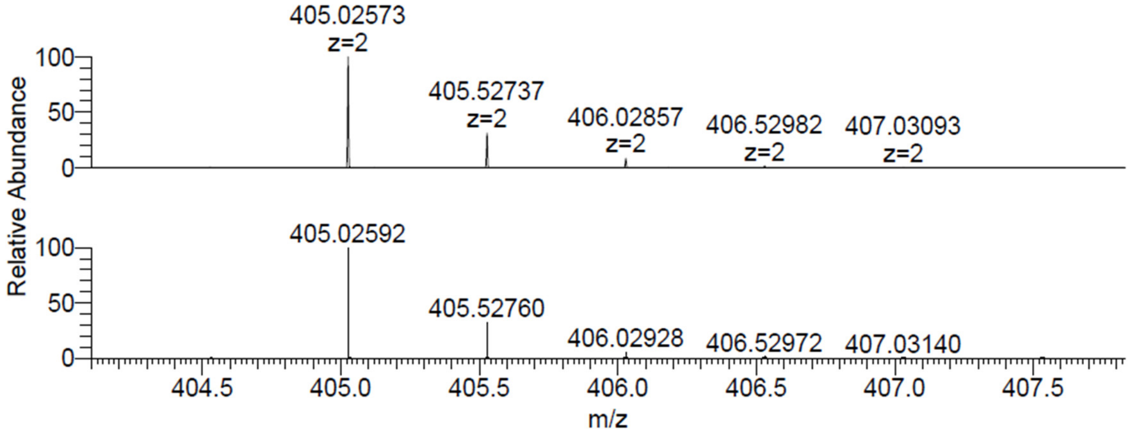

Figure S15. Experimental (top) and calculated (bottom) isotopic pattern for the peak of $[(V^{IV}O)_2(L4)_2+2H]^{2+}$ detected in the ESI-MS(+) spectrum of the system $V^{IV}O^{2+}$-L4 at 1:1 molar ratio (LC-MS H_2O, ligand concentration 50 μM).

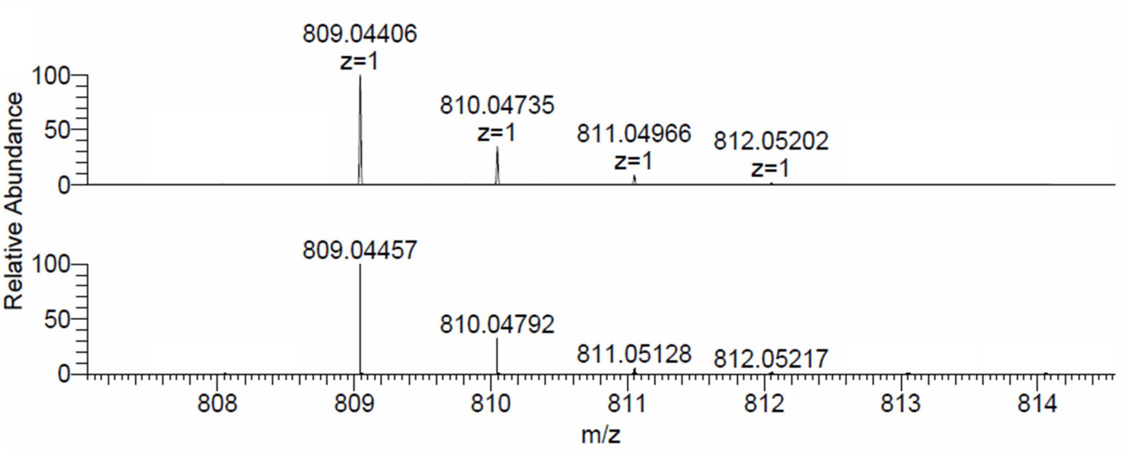

Figure S16. Experimental (top) and calculated (bottom) isotopic pattern for the peak of $[(V^{IV}O)_2(L4)_2+H]^+$ detected in the ESI-MS(+) spectrum of the system $V^{IV}O^{2+}$-L4 at 1:1 molar ratio (LC-MS H_2O, ligand concentration 50 μM).

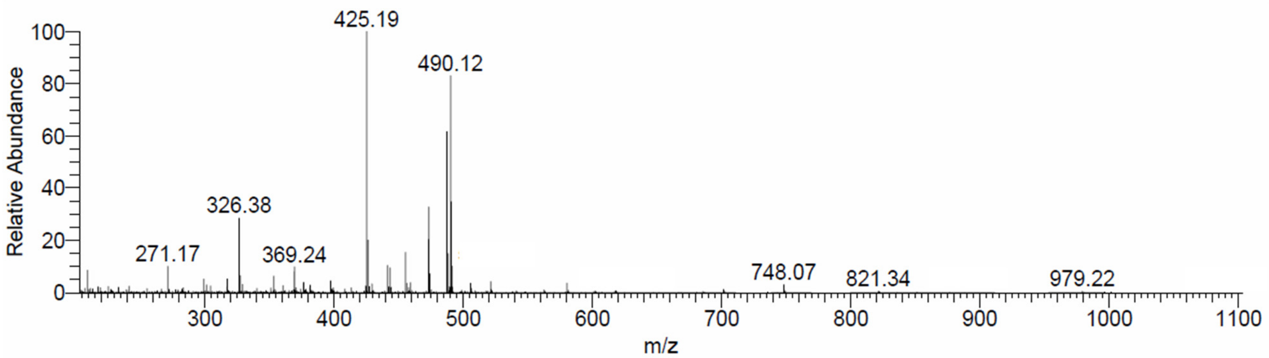

Figure S17. ESI-MS(+) spectrum recorded on the system $V^{IV}O^{2+}$-L9 at 1:1 molar ratio (LC-MS MeOH, ligand concentration 5 μM).

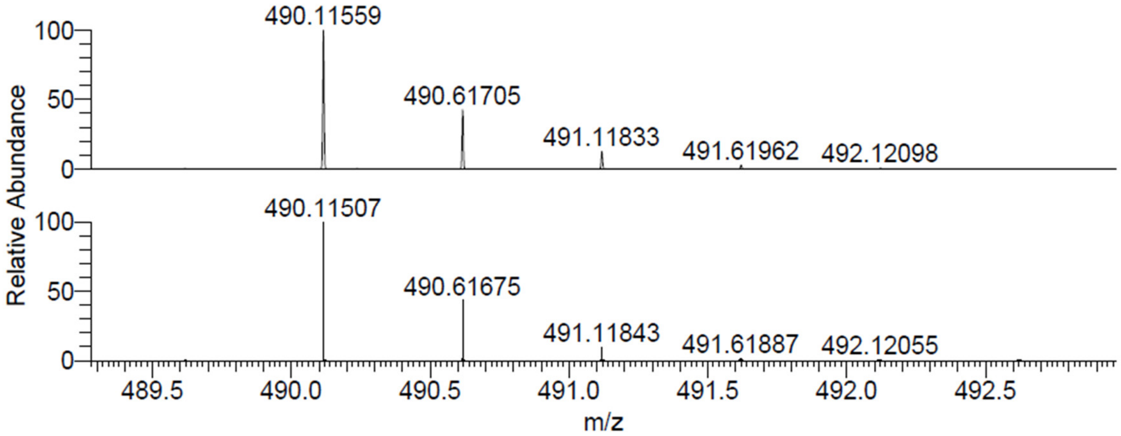

Figure S18. Experimental (top) and calculated (bottom) isotopic pattern for the peak of $[(V^{IV}O)_2(L9)_2+2H]^{2+}$ detected in the ESI-MS(+) spectrum of the system $V^{IV}O^{2+}$-L9 at 1:1 molar ratio (LC-MS MeOH, ligand concentration 5 μM).

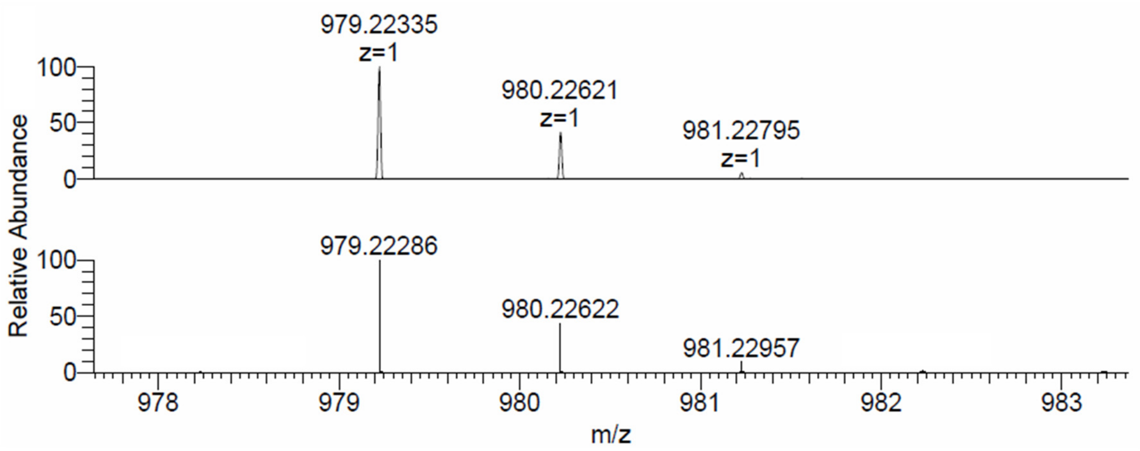

Figure S19. Experimental (top) and calculated (bottom) isotopic pattern for the peak of $[(V^{IV}O)_2(L9)_2+H]^+$ detected in the ESI-MS(+) spectrum of the system $V^{IV}O^{2+}$-L9 at 1:1 molar ratio (LC-MS MeOH, ligand concentration 5 μM).

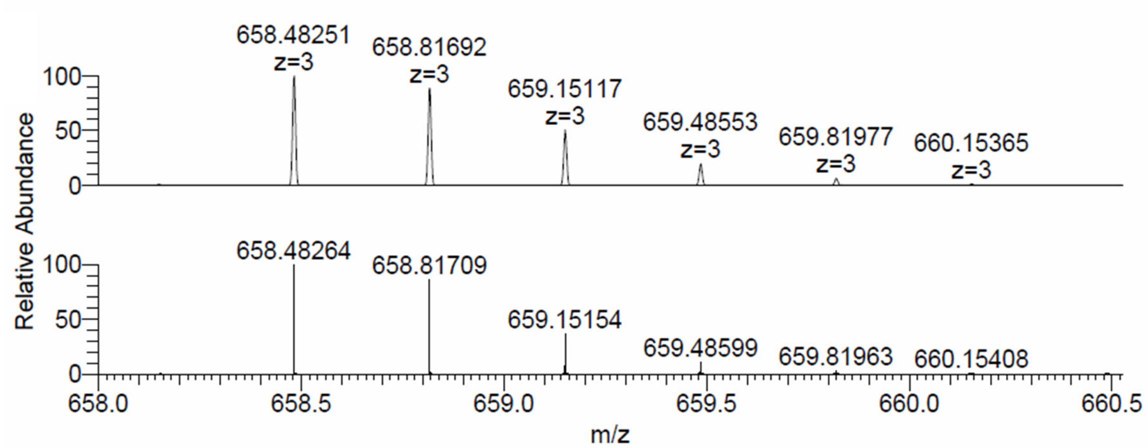

Figure S20. Experimental (top) and calculated (bottom) isotopic pattern for the peak of $[(V^{V}{}_2O_3)(V^{IV}O_2)_2(L9)_4+3H]^{3+}$ detected in the ESI-MS(+) spectrum of the system $V^{IV}O^{2+}$-L9 at 1:1 molar ratio (LC-MS H_2O, ligand concentration 50 μM).

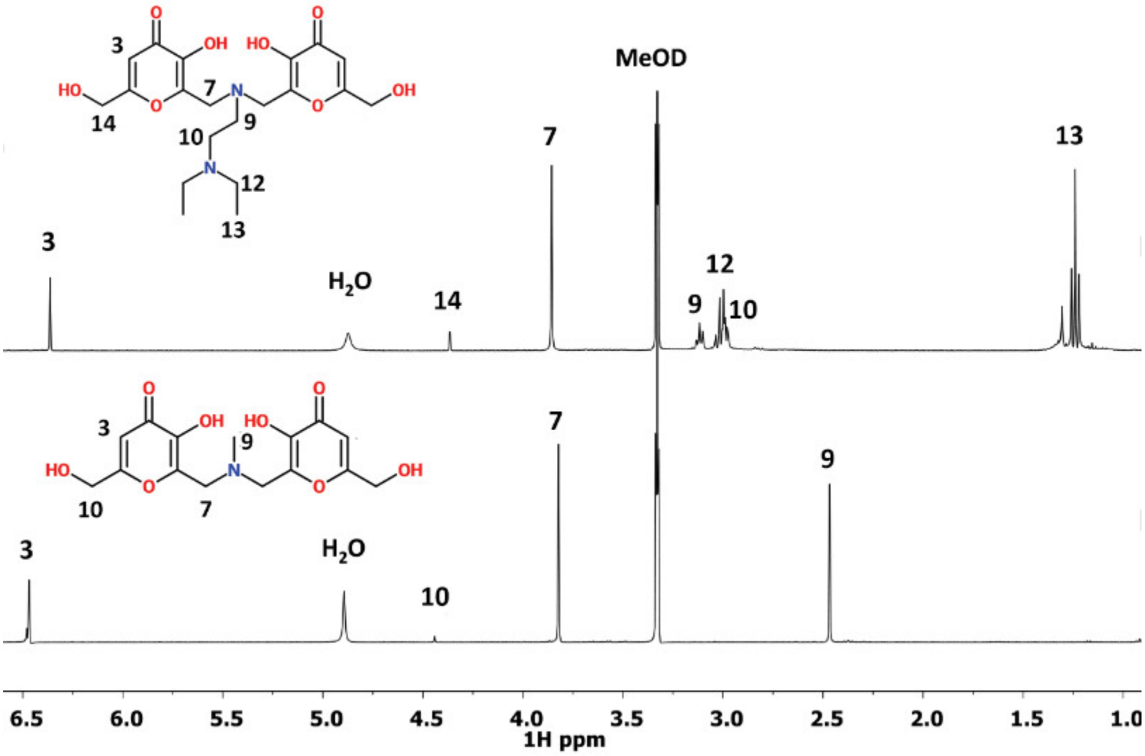

Figure S21. 1D ^{1}HNMR spectra of free ligands L4 (bottom) and L9 (top) in MeOD.

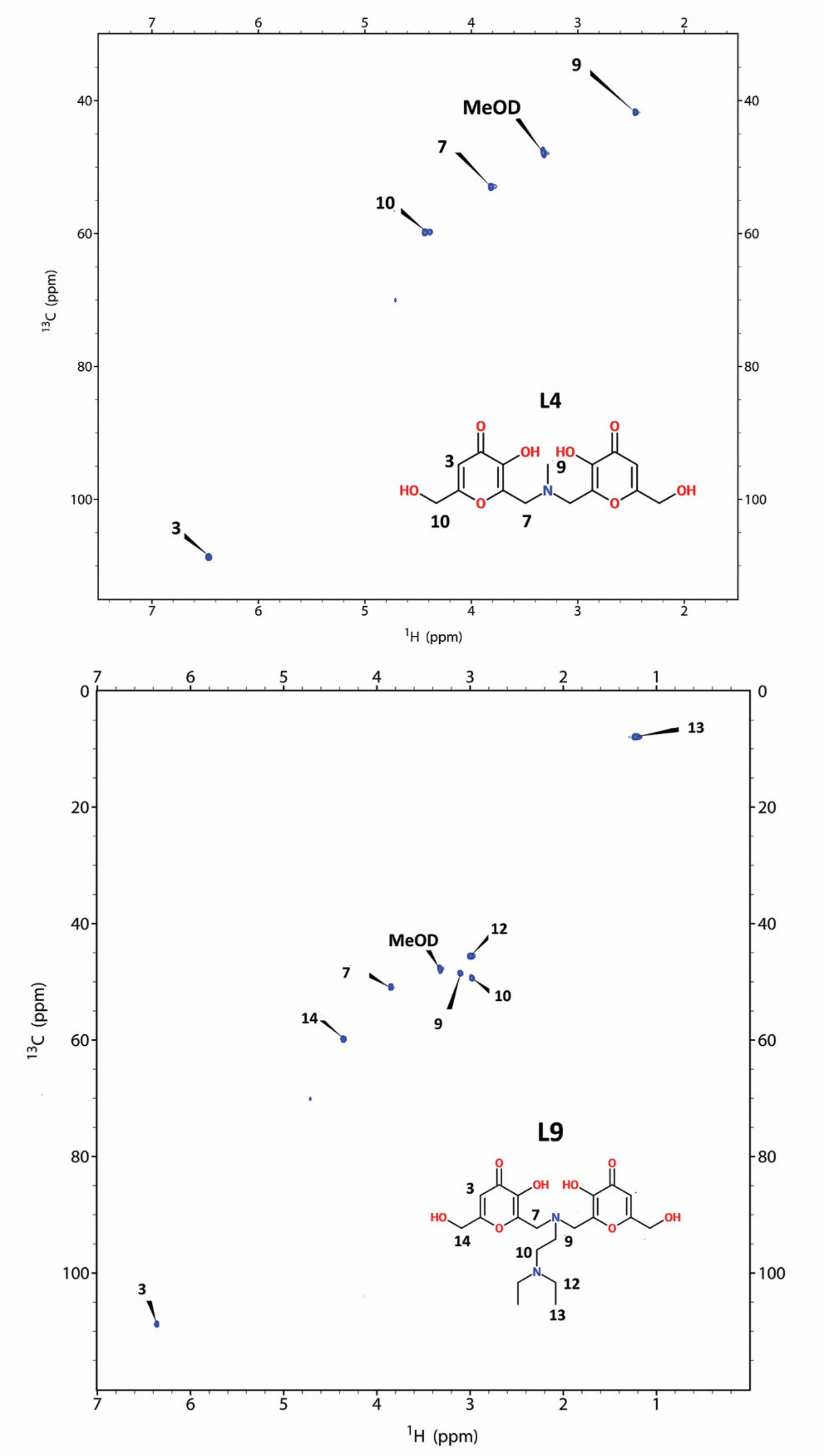

Figure S22. NMR HSQC of L4 and L9 ligands in MeOD.

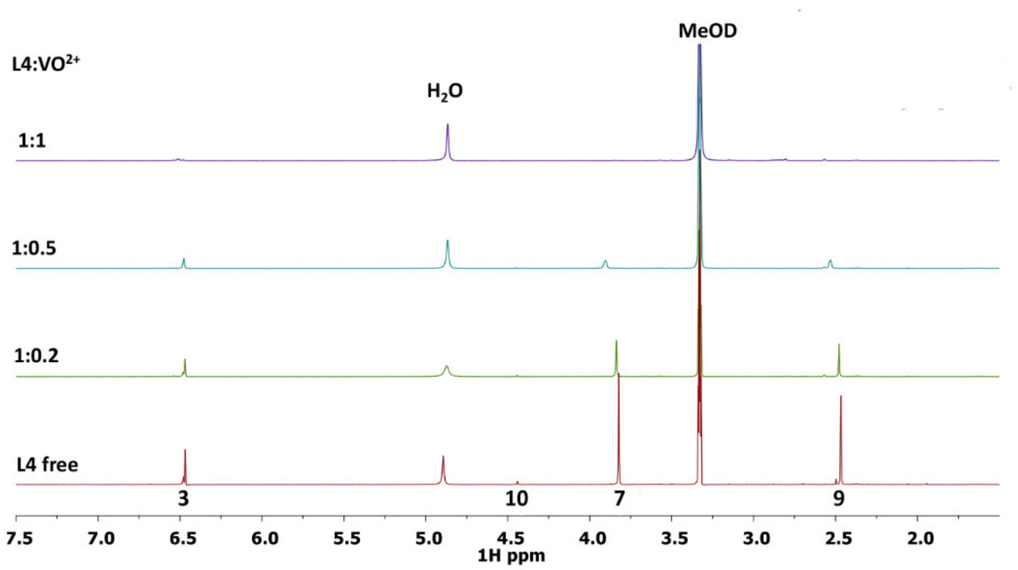

Figure S23. 1D ^{1}HNMR spectra of L4-V^{IV}O^{2+} system in MeOD at different L4:V^{IV}O^{2+} ratios.

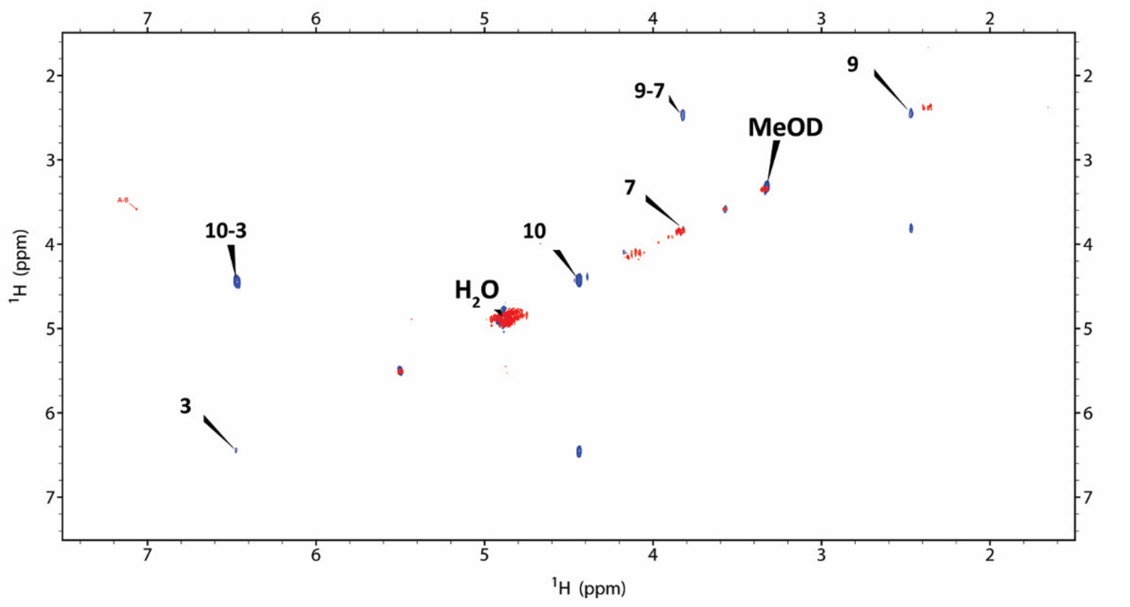

Figure S24. Comparison of 2D ^{1}H-^{1}H NMR COSY spectra of L4 free (blue) and L4-V^{IV}O^{2+} (red) systems in MeOD solution.
